# Quantitative assessment of the effect of pre-gestational diabetes and risk of adverse maternal, perinatal and neonatal outcomes

**DOI:** 10.18632/oncotarget.17824

**Published:** 2017-05-11

**Authors:** Lei Yu, Xiao-Ling Zeng, Ming-Liang Cheng, Guo-Zhen Yang, Bi Wang, Zi-Wen Xiao, Xin Luo, Bao-Fang Zhang, De-Wei Xiao, Shuai Zhang, Hua-Juan Liu, Ya-Xin Hu, Hou-Kang Lei, Qin-Fen Li, Zheng-Rong Wang

**Affiliations:** ^1^ Prenatal Diagnosis Center, Hospital Affiliated to Guizhou Medical University, Guiyang 550004, Guizhou, China; ^2^ The First Affiliated Hospital of Jinan University, Guangdong 510632, Guangzhou, China; ^3^ Department of Infectious Diseases, Hospital Affiliated to Guizhou Medical University, Guiyang 550004, Guizhou, China; ^4^ Department of Eugenics and Genetics, Guiyang Maternal and Child Health-Care Hospital, Guiyang 550003, Guizhou, China; ^5^ Department of Interventional Radiology, Cancer Hospital of Guizhou Medical University, Guiyang 550004, Guizhou, China

**Keywords:** pre-gestational diabetes, adverse pregnancy outcomes, risk, meta-analysis

## Abstract

Pregnancies complicated by pre-gestational diabetes (PGD) are associated with a higher rate of adverse outcomes, including an increased rage of preterm delivery, pregnancy-induced hypertension, pre-eclampsia, caesarean section, perinatal mortality, stillbirth, shoulder dystocia, macrosomia, small for gestational age, large for gestational age, low birth weight, neonatal hypoglycemia, neonatal death, low Apgar score, NICU admission, jaundice and respiratory distress. In the past two decades, numerous reports have been published regarding associations between PGD and risk of adverse outcomes. However, study results are inconsistent. To provide a synopsis of the current understanding of PGD for risk of adverse pregnancy outcomes, a random-effects meta-analysis over 40 million subjects from 100 studies was performed to calculate the pooled ORs. Potential sources of heterogeneity were systematically explored by multiple strata analyses and meta-regression. Overall, PGD were significantly associated with increased risk of preterm delivery (OR=3.48), LGA (OR=3.90), perinatal mortality (OR=3.39), stillbirth (OR=3.52), pre-eclampsia (OR=3.48), caesarean section (OR=3.52), NICU admission (OR=3.92), and neonatal hypoglycemia (OR=26.62). Significant results were also observed for 7 adverse outcomes with OR range from 1.54 to 2.82, while no association was found for SGA and respiratory distress after Bonferroni correction. We found that women with T1DM had higher risks for most of adverse pregnancy outcomes compared with women with T2DM. When stratified by study design, sample size, type of diabetes, geographic region, and study quality, significant associations remains. Our findings demonstrated that PGD is a strong risk-conferring factor for adverse maternal, perinatal and neonatal outcomes.

## INTRODUCTION

Diabetes is one of the most common pre-existing maternal disorders and complicated approximately 1.3% of all pregnancies [1, 2]. Most women with pre-gestational diabetes (PGD) characterised by disturbance in glucose metabolism may be due to variable degrees of insulin resistance (type 2), or a consequence of autoimmune destruction of the pancreatic β-cells (type 1). With increasing numbers type 1 diabetes diagnosed among youth and high prevalence of obesity among women of child-bearing age [3–5], the demographic pattern of PGD is changing. Besides, the sex ratio of newly diagnosed type 2 diabetes among youth was remarkably skewed to female [6–8]. Therefore, continuous increase in diabetes rates in the global population will translate into higher prevalence of PGD eventually [9].

Women with PGD are associated with adverse pregnancy outcomes. Unfortunately, the goal of the St. Vincent Declaration has not yet been achieved despite intensive maternal and neonatal care [10]. Furthermore, PGD has also been associated with increased risk of maternal complications including shoulder dystocia, gestational hypertension, and pre-eclampsia [11–13], making pre-pregnancy care particularly glycaemic control and obstetrical interventions of great importance. More recent studies on PGD have reported various results: preterm delivery and stillbirth were still significantly increased in some studies [2, 14, 15] despite good metabolic control, while other studies [16–18] reported that PGD was no longer a significant factor. These conflicting results may be partly due to ethnic diversity, insufficient power, phenotypic heterogeneity, and even publication biases [19]. Until now, no quantitative assessment has focused on PGD with the risk various adverse pregnancy outcomes. Therefore, we conducted a comprehensive meta-analysis on all eligible studies to clarify this inconsistency and to establish a comprehensive picture of the relationship.

## RESULTS

### Characteristics of the included studies

**Figure 1:**
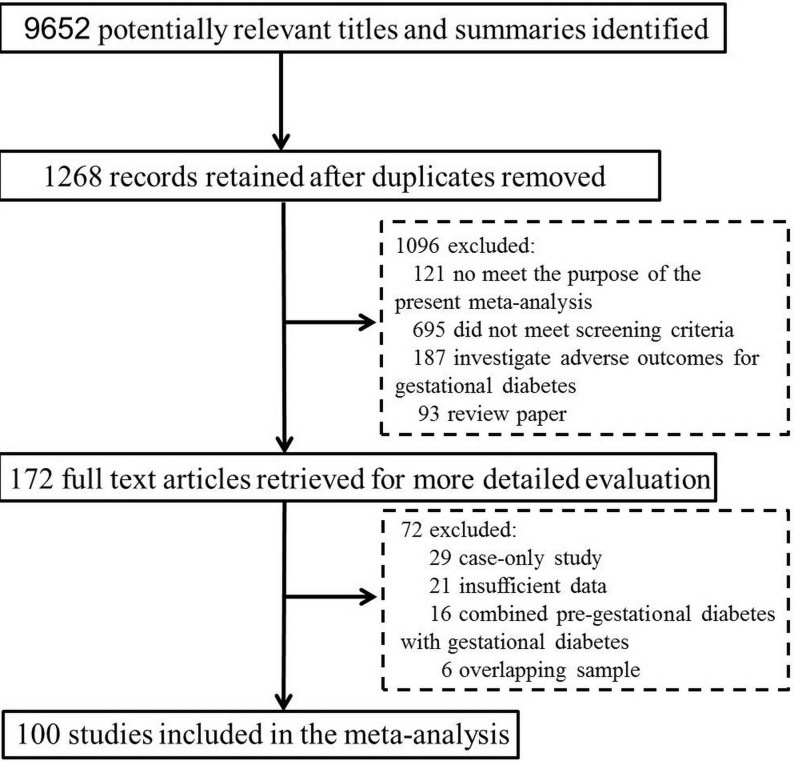
Flow diagram for selection and inclusion of studies

A detailed flowchart of literature search is shown in Figure 1. We identified a total of 9650 citations from the databases and 2 from reference lists. After exclusion of duplicate reports and studies that did not meet our inclusion criteria, a total of 100 publications were finally included in this study. Main characteristics of the included studies were summarised in Supplementary Table 1. These eligible studies were published between 1993 and 2017, and included a total of 304,144 women with PGD and > 40 million non-diabetic populations. Almost all included studies were conducted in developed country, including 28 studies in the North American, 48 in Europe, 3 in East Asian, 11 in Oceania, 3 in Africa and 7 with subjects recruited from Middle East. 62 summarized results by two-by-two tables, whereas 38 reported adjusted risk estimates. As for methodological quality assessment, 47 studies were awarded ≥ 7 points, and 30 studies were awarded 5 to 6 points, indicating that the quality of included studies were of median-to-high quality. Almost all included studies recruited women with singleton pregnancy, and only two studies included women with twin pregnancy.

### Quantitative data synthesis

**Figure 2:**
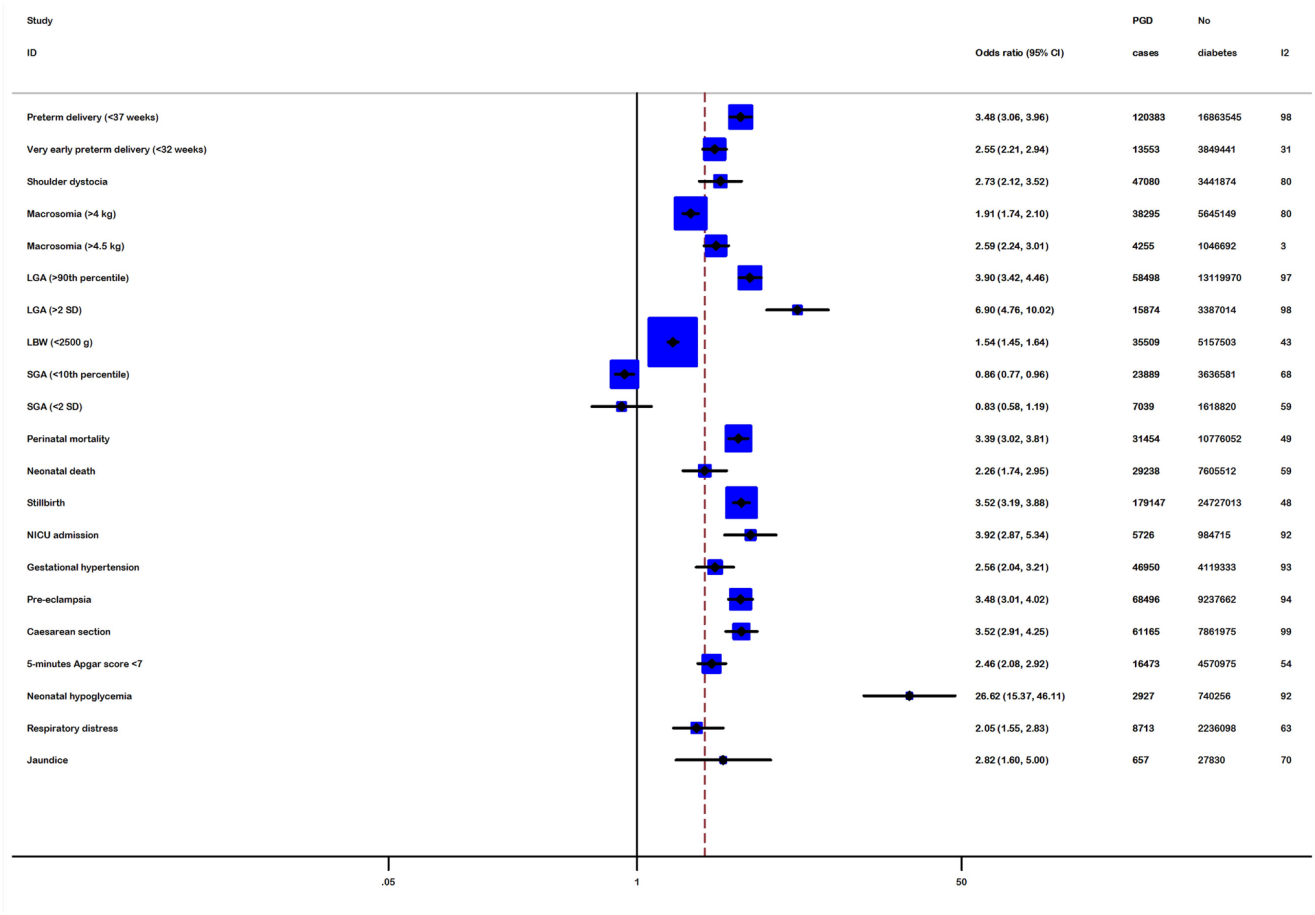
Meta-analysis with a random-effect model for the association between pre-gestational diabetes and adverse pregnancy outcomes

Figure 2 shows the summary OR estimates for adverse pregnancy outcomes comparing women with PGD with non-diabetic population.

The adverse maternal outcomes were preterm delivery, very early preterm delivery, shoulder dystocia, caesarean section, pregnancy-induced hypertension (PIH) and pre-eclampsia. The risk of preterm delivery at or before 37 weeks was 3.48 (95%CI: 3.06-3.96, P<10^-5^), whereas the risk was 2.55 (95%CI: 2.21-2.94, P<10^-5^) for very early preterm delivery in women with PGD. There was significant difference in maternal hypertensive complications. Using a random-effects model, the pooled OR of PIH was OR=2.56 (95%CI: 2.04-3.21, P<10^-5^). In addition, mother with PGD have statistically significant association with pre-eclampsia (OR=3.48; 95%CI: 3.01-4.02, P<10^-5^). Furthermore, PGD conferred much higher risk of requiring caesarean section (OR=3.52; 95%CI: 2.91-4.25, P<10^-5^). In vaginal deliveries, the risk of shoulder dystocia occurred in infants delivered by pre-gestational diabetic mothers was much higher than control infants with an OR of 2.73 (95%CI: 2.12-3.52, P<10^-5^).

As for perinatal outcomes including macrosomia, LGA, SGA, LBW, stillbirth, perinatal mortality, and low Apgar scores, the risk for women with PGD giving birth to LGA babies was significantly higher than for women without diabetes (OR=3.90; 95%CI: 3.42-4.46, P<10^-5^). Similarly, women with PGD were more likely to have a macrosomic infant (OR=1.91; 95%CI: 1.74-2.10, P<10^-5^) and low birth weight babies than controls (OR=1.54; 95% CI: 1.45-1.64, P<10^-5^). In contrast, the risk of delivering an SGA infant was 0.86 (95%CI: 0.77-0.96, P=0.007). Women with PGD were more likely to have stillbirth (OR=3.52; 95%CI: 3.19-3.88, P<10^-5^); thus, perinatal mortality (OR=3.39; 95%CI: 3.02-3.81, P<10^-5^) risks for infants of PGD mothers were also significantly higher than in the nondiabetic population (Table 1).

**Table 1:** Associations of pre-gestational diabetes and adverse pregnancy outcomes

Outcomes	No. of datasets	Overall			No. of datasets	Cohort based study			No. of datasets	Non-Cohort based study		
		OR (95%CI)	P(Z)	P(Q)		OR (95%CI)	P(Z)	P(Q)		OR (95%CI)	P(Z)	P(Q)
Preterm delivery (< 37 weeks)	55	3.48 (3.06-3.96)	<10^-5^	<10^-5^	21	3.69 (3.12-4.36)	<10^-5^	<10^-5^	34	3.36 (2.85-3.96)	<10^-5^	<10^-5^
Very early preterm delivery (< 32 weeks)	4	2.55 (2.21-2.94)	<10^-5^	0.22	4	2.55 (2.21-2.94)	<10^-5^	0.22	NA	NA	NA	NA
Macrosomia (> 4 kg)	17	1.91 (1.74-2.10)	<10^-5^	<10^-5^	6	1.98 (1.42-2.76)	<10^-4^	<10^-4^	11	1.92 (1.77-2.08)	<10^-5^	0.002
Macrosomia (> 4.5 kg)	4	2.59 (2.24-3.01)	<10^-5^	0.38	1	2.20 (1.56-3.11)	<10^-5^	NA	3	2.70 (2.32-3.14)	<10^-5^	0.38
LGA (>90^th^ percentile)	35	3.90 (3.42-4.46)	<10^-5^	<10^-5^	16	3.79 (3.20-4.48)	<10^-5^	<10^-5^	19	3.96 (3.22-4.87)	<10^-5^	<10^-5^
LGA (> 2 SD)	6	6.90 (4.76-10.02)	<10^-5^	<10^-5^	3	8.06 (5.87-11.06)	<10^-5^	<10^-5^	3	5.94 (3.99-8.85)	<10^-5^	<10^-5^
LBW (<2500 g)	12	1.54 (1.45-1.64)	<10^-5^	0.06	2	1.67 (1.46-1.91)	<10^-5^	0.33	9	1.51 (1.42-1.62)	<10^-5^	0.06
SGA (<10^th^ percentile)	20	0.86 (0.77-0.96)	0.007	<10^-5^	13	0.89 (0.78-1.00)	0.06	<10^-5^	7	0.81 (0.67-0.98)	0.03	0.09
SGA (< 2 SD)	4	0.83 (0.58-1.19)	0.32	0.06	2	0.89 (0.54-1.48)	0.65	0.05	2	0.69 (0.28-1.71)	0.42	0.07
Perinatal mortality	27	3.39 (3.02-3.81)	<10^-5^	<10^-4^	11	3.67 (3.29-4.09)	<10^-5^	0.72	16	3.25 (2.70-3.92)	<10^-5^	<10^-4^
Neonatal death	19	2.26 (1.74-2.95)	<10^-5^	0.001	7	2.46 (1.99-3.05)	<10^-5^	0.70	12	2.28 (1.46-3.57)	<10^-4^	<10^-5^
Stillbirth	39	3.52 (3.19-3.88)	<10^-5^	<10^-4^	19	3.85 (3.33-4.45)	<10^-5^	0.03	20	3.19 (2.87-3.55)	<10^-5^	0.19
PIH	21	2.56 (2.04-3.21)	<10^-5^	<10^-5^	11	2.27 (1.65-3.14)	<10^-5^	<10^-5^	10	2.85 (2.06-3.94)	<10^-5^	<10^-5^
Pre-eclampsia	48	3.48 (3.01-4.02)	<10^-5^	<10^-5^	30	3.59 (3.11-4.14)	<10^-5^	<10^-5^	18	3.35 (2.52-4.45)	<10^-5^	<10^-5^
Caesarean section	42	3.52 (2.91-4.25)	<10^-5^	<10^-5^	16	3.52 (2.69-4.61)	<10^-5^	<10^-5^	26	3.51 (2.81-4.37)	<10^-5^	<10^-5^
Shoulder dystocia	16	2.73 (2.12-3.52)	<10^-5^	<10^-5^	7	2.38 (1.92-2.95)	<10^-5^	0.08	9	2.82 (1.73-4.60)	<10^-4^	<10^-5^
NICU admission	14	3.92 (2.87-5.34)	<10^-5^	<10^-5^	4	3.47 (0.78-15.37)	0.10	<10^-5^	10	3.95 (2.93-5.34)	<10^-5^	<10^-5^
5-minutes Apgar score < 7	13	2.46 (2.08-2.92)	<10^-5^	0.01	5	2.32 (1.86-2.91)	<10^-5^	0.33	8	2.58 (2.03-3.27)	<10^-5^	0.004
Neonatal hypoglycemia	11	26.62 (15.37-46.11)	<10^-5^	<10^-4^	3	8.06 (4.51-14.41)	<10^-5^	0.09	8	54.59 (49.42-60.30)	<10^-5^	0.56
Respiratory distress	8	2.09 (1.55-2.83)	0.003	0.009	3	2.09 (0.80-5.43)	0.13	0.003	5	2.00 (1.56-2.55)	0.001	0.003
Jaundice	6	2.82 (1.60-5.00)	<10^-4^	0.005	2	3.66 (1.88-7.13)	<10^-4^	0.04	4	2.28 (1.00-5.22)	0.05	0.09

As for secondary neonatal outcomes, women with PGD were more likely than those of women without diabetes to have neonatal hypoglycaemia (OR=26.62; 95%CI: 15.37-46.11, P<10^-5^), neonatal death (OR=2.26; 95%CI: 1.74-2.95, P<10^-5^), jaundice (OR=2.82; 95%CI: 1.60-5.00, P<10^-4^) and to be admitted to an NICU (OR=3.92; 95%CI: 2.87-5.34, P<10^-5^). Similarly, the results showed a significant association of PGD mothers with increased risk of low 5-minute Apgar score (OR=2.46; 95%CI: 2.08-2.92, P<10^-5^), while the risk of delivering an infant with respiratory distress was 2.09 (95%CI: 1.55-2.83, P=0.003).

The association for 19 adverse outcomes were still highly significant, but SGA and respiratory distress were failed to pass Bonferroni correction for multiple testing in 21 outcomes.

### Sources of heterogeneity

To explore the heterogeneity among studies of women with PGD and adverse pregnancy outcomes, we performed stratified analyses and meta-regression. The significant associations between PGD and risk of adverse pregnancy outcomes in almost all subgroup analyses defined by study design (Table 1), sample size, geographic region, and study quality (Supplementary Tables 2-4). To minimize bias from confounding, we limited the analysis to studies reporting adjusted estimates only, and results after adjustment appeared to have lower estimates than those not adjusting (Supplementary Table 5), although the results did not materially change. Some studies reported outcome data on women with PGD separating T1DM and T2DM. In comparison with women with T2DM, the association was somewhat stronger in women with T1DM for most of adverse pregnancy outcomes (Table 2). Meta-regression analyses were then conducted and find there was little evidence of heterogeneity between any of these subgroups (Supplementary Table 6).

**Table 2:** Associations of pre-gestational diabetes and adverse pregnancy outcomes stratified by type of diabetes

Outcomes	No. of datasets	T1DM			No. of datasets	T2DM		
		OR (95%CI)	P(Z)	P(Q)		OR (95%CI)	P(Z)	P(Q)
Preterm delivery (< 37 weeks)	15	4.36 (3.72-5.12)	<10^-5^	<10^-5^	10	2.96 (2.13-4.10)	<10^-5^	<10^-4^
Macrosomia (> 4 kg)	5	2.10 (1.61-2.74)	<10^-5^	0.001	4	1.33 (0.82-2.17)	0.25	0.01
LGA (> 90^th^ percentile)	8	4.61 (2.83-7.53)	<10^-5^	<10^-5^	7	2.32 (1.48-3.64)	<10^-4^	<10^-4^
SGA (< 10^th^ percentile)	7	0.68 (0.56-0.83)	<10^-4^	0.47	7	1.34 (0.98-1.82)	0.07	0.28
Perinatal mortality	14	3.80 (3.14-4.60)	<10^-5^	0.004	5	3.63 (2.84-4.65)	<10^-5^	0.36
Neonatal death	8	2.73 (2.13-3.49)	<10^-5^	0.44	3	3.57 (1.87-6.80)	<10^-4^	0.37
Stillbirth	12	3.97 (3.44-4.58)	<10^-5^	0.70	4	3.65 (1.59-8.42)	0.002	0.14
PIH	7	2.68 (1.85-3.89)	<10^-5^	<10^-5^	6	1.66 (1.04-2.66)	0.03	0.03
Pre-eclampsia	10	4.19 (3.08-5.71)	<10^-5^	<10^-5^	6	1.86 (0.99-3.51)	0.06	<10^-4^
Caesarean section	17	3.97 (3.31-4.77)	<10^-5^	<10^-5^	8	2.40 (1.79-3.21)	<10^-5^	<10^-4^
Shoulder dystocia	4	4.49 (2.16-9.30)	<10^-5^	0.001	4	2.01 (1.21-3.34)	0.007	0.46
NICU admission	4	3.95 (1.70-9.18)	0.001	<10^-4^	3	5.29 (1.13-24.66)	0.03	<10^-4^
5-minutes Apgar score < 7	5	2.55 (2.17-3.00)	<10^-5^	0.55	2	1.18 (0.49-2.85)	0.72	0.46
Neonatal hypoglycemia	5	33.53 (15.54-72.32)	<10^-5^	<10^-5^	4	7.73 (3.82-15.65)	<10^-5^	0.27
Respiratory distress	4	2.50 (1.86-3.37)	<10^-5^	0.22	2	1.25 (0.59-2.63)	0.56	0.04
Jaundice	3	3.13 (0.84-11.69)	0.09	0.001	3	2.53 (1.46-4.38)	0.001	0.29

### Sensitivity analysis and publication bias

To assess the extent to which individual studies with extremely large ORs influenced the summary OR, we carried out a leave-one-out sensitivity analysis, in which one study at a time was excluded and the remaining were re-analysed. The results indicated that no individual study influenced the pooled OR qualitatively, suggesting robustness of the meta-analyses (Supplementary Figure 1). The visual inspection of funnel plots for adverse pregnancy outcomes and women with PGD displayed a symmetrical distribution (Supplementary Figure 2) and there was no evidence of small study effects with Egger’s test or with Begg’s test (P > 0.1 for all, Supplementary Table 7).

## DISCUSSION

As the incidence of type 2 diabetes and obesity continues to increase, diabetes in pregnancy has emerged as a growing concern. Numerous of studies have investigated outcomes for women with PGD and reported inconsistent results. The current study is, as far as we know, the first meta-analysis confirmed that PGD, after adjustment for multiple outcomes, is associated with increased risk for a wide range of adverse pregnancy outcomes, including pregnancy-induced hypertension, pre-eclampsia, preterm delivery, macrosomia, LGA, low birthweight, stillbirth, perinatal mortality, neonatal death, neonatal hypoglycemia, jaundice, low 5-minute Apgar score, and caesarean section, but not SGA and respiratory distress. These complications have important implications for obstetrical outcomes, highlighting the importance of glycemic control.

Macrosomia or LGA remains a major perinatal concern for women with PGD [20]. High maternal glucose levels stimulated foetal insulin secretion and foetal overgrowth [21], delayed fetal lung maturation and neonatal hypoglycaemia [22]. LGA and macrosomia are also associated with short term health complications [23, 24] like shoulder dystocia, higher risk of caesarean section, and long term obesity and dysglycemia [25]. Even by optimal diabetes control, however, macrosomia may not be completely preventable in the 3rd trimester of pregnancy [26].

As stillbirths account for approximately 60% of perinatal mortality [27], we observed significantly increased risk of stillbirth and perinatal mortality on the PGD women compared with the non-diabetic. Accumulative evidences have suggested that chronic fetal hypoxia was the leading causes of stillbirth [28, 29], while maternal and fetal hyperglycemia was suggested as an etiology for antenatal asphyxia [30]. Preterm delivery was 3.4 times that of the control deliveries, which also contribute to neonatal morbidity and higher incidence of secondary neonatal outcomes such as admission to NICU, jaundice, low Apgar score. Therefore, a combination of identify the populations at risk with prenatal services and obstetric interventions is essential to reduce the incidence of stillbirths and perinatal mortality.

The results of the current study showed a considerable increased risk of hypertensive complications among women with PGD. Indeed, there is a direct relationship between abnormal glucose metabolism before pregnancy and the development of preeclampsia [31, 32], and the risk increases with the severity of the metabolic disturbance [33]. Furthermore, previous studies have reported that superimposed preeclampsia in women with PGD further increases the risk of adverse birth outcomes, including preterm birth, macrosomia, and delivery by caesarean section [34, 35], which influence stillbirths and neonatal deaths.

In sub-group analysis by study design, geographic region, sample size, adjustment, and study quality, consistent association results were observed although significant between-study heterogeneity remain. The presence of heterogeneity can result from infant sex, gestational age, alcohol use, smoking habits, obesity, previous obstetric history, glycaemic control and duration of diabetes exposure.

Glycemic disturbance is usually more severe in pregnant women with T1D than in those with T2D as difference in duration of diabetes exposure. Our results suggested that T1D has a greater impact compared with T2D in terms of adverse pregnancy outcomes, which was in general agreement with findings of previously published work [36]. In fact, several studies have reported a linear relationship between maternal glucose levels and adverse pregnancy outcomes [20, 37]. Besides, increased duration of diabetes has been associated with increase the risk of adverse pregnancy outcomes including preeclampsia [35], stillbirth [38]. Thus, adverse pregnancy outcomes might be more prevalent in women with T1D than in those with T2D.

The strength of this study lies in the fact that it summarizes adverse pregnancy outcomes in women with PGD based on > 40 million deliveries worldwide during more than 20 years. Different outcomes, study design, geographic regions, were represented and potential source of heterogeneity were systematically explored. Despite its originality and robust size, our study has several limitations. As failure to adjust for matching factors, multiple strata analyses were performed based on a fraction of all available data to be pooled. This may have introduced a selection bias and the results may be overinflated [39]. We were not able to obtain individual-level data, whereas a more precise analysis with adjustment of confounding factors could be conducted. However, the overall results pooled from adjusted estimate were in general agreement with those calculated from raw data. As most of studies have recruited individual from white, and studies of other ethnicities may have been underpowered. As differences in genetic background, life style and diabetes prevalence, studies with large sample size of East Asians or Africans are warranted to further validate results of present study.

In summary, our meta-analysis of 100 studies indicates that PGD may increase the risks of adverse maternal, perinatal and neonatal outcomes. As PGD becomes more common, there is a growing need for preventive measures including continuous glucose monitoring, dietary advice, and assessment of risk indicators to prevent development of adverse outcomes.

## MATERIALS AND METHODS

The outcomes of interest were PGD related 21 type of adverse pregnancy outcomes including pregnancy-induced hypertension (PIH), pre-eclampsia (PE), preterm delivery (PTD, defined as prior to 37 completed weeks of gestation), very early PTD (VEPTD, defined as birth at < 32 weeks of gestation), macrosomia (defined as birthweight > 4000 g or > 4500 g), large for gestational age (LGA, defined as birthweight above the 90^th^ percentile or 2 SD above the mean for normal fetal growth), small for gestational age (SGA, defined as birthweight below the 10^th^ percentile or 2 SD below the mean for normal fetal growth), low birthweight (LBW, defined by birth weight of < 2500 g), admission to neonatal intensive care unit (NICU), stillbirth (defined as intrauterine fetal death after 20 weeks’ gestation), perinatal mortality (defined as stillbirth or death within the first 7 days of life), neonatal death (defined as death of a live born infant within the first 28 days of life), neonatal hypoglycemia, jaundice, respiratory distress, caesarean section, and low Apgar scores (defined as a score of less than 7 at 5 minutes after delivery).

### Identification of relevant studies

We conducted a systematic computer-based search from databases including PubMed, ISI Web of Science, Cochrane Library, Embase and SCOPUS without language restriction. Search was limited to human studies, restricting our query to publications from January 1990 to February 2017. Search strategies were keywords relating PGD (pre-gestational diabetes OR pre-pregnancy diabetes OR type 1 diabetes OR insulin dependent diabetes mellitus OR type 2 diabetes OR non-insulin dependent diabetes mellitus) in combination with words related to various adverse pregnancy outcomes. In addition, references from retrieved studies were checked by a manual search for additional eligible studies. MOOSE (meta-analysis of observational studies in epidemiology) guidelines were followed in the present study [40].

### Selection criteria and quality assessment

Studies were included if they fulfilled all of the following criteria: (1) providing evidence one women with PGD and risk of adverse pregnancy outcomes using cohort or case-control design; (2) original research with independent data; (3) with sufficient data to calculate odds ratio (OR) with its 95% confidence intervals (CIs) and P value, (4) identification of PGD patients was confirmed pathologically. The major reasons for exclusion of studies were (1) studies combing PGD with gestational diabetes, (2) case-only studies, (3) review paper, and (4) non-peer-reviewed articles. If the same population was reported in different studies, the study with the longest follow-up time or the most information was included. For included studies, a quantitative quality score according to Newcastle–Ottawa Scale was adapted to assess study methodology [41].

### Data extraction

Relevant data was extracted independently by two reviewers. For each qualified study, the following variables were collected: the first author, published year, country, ethnicity, study design, identification of cases and controls, sample size, maternal obesity, mother’s age at birth, body mass index (BMI), duration of diabetes, type of pre-gestational diabetes (T1DM, T2DM), nulliparous, plurality (singleton, twins, high order multiple pregnancies), cigarette smoking, preconception care, maternal and perinatal outcomes. Where available, adjusted estimates with the most stringent adjustment for confounding variables were extracted. Other-wise, unadjusted ORs were calculated from the raw data presented in the paper. Disagreement in the review reports was resolved by further discussion between authors through consensus.

### Statistical analysis

Since adverse pregnancy outcome of women with PGD is a relatively rare, Hazard ratio (HR), relative risk (RR) and rate ratio were treated as equivalent estimates of OR [42]. Random-effects model taking into account between-study variation [43] was used to compute a pooled OR. To assess qualitatively and quantitatively between-study heterogeneity, we calculated the Cochran Q and I^2^ statistics [44]. Subgroup analyses and meta-regression (i.e., study design, sample size, and geographic region) were performed to investigate potential sources of heterogeneity. Small-study effects, such as publication bias, was assessed using Egger’s test [45] and Begg’s test [46], with P < 0.10 considered significance due to limited power of the test. Sensitivity analyses were performed to explore possible explanations for heterogeneity, and to assess the stability of the results. All analyses were performed using the Stata software (Version 10.1; STATA Corp., College Station, TX, USA). Bonferroni correction was used to adjust our results for multiple outcomes testing (0.05/21 = 0.0024). Statistical significance was defined as two-sided at the P = 0.05 level.

## SUPPLEMENTARY MATERIALS FIGURES AND TABLES





## References

[1] Lawrence JM, Contreras R, Chen W, Sacks DA (2008). Trends in the prevalence of preexisting diabetes and gestational diabetes mellitus among a racially/ethnically diverse population of pregnant women, 1999-2005. Diabetes Care.

[2] Shand AW, Bell JC, McElduff A, Morris J, Roberts CL (2008). Outcomes of pregnancies in women with pre-gestational diabetes mellitus and gestational diabetes mellitus; a population-based study in New South Wales, Australia, 1998-2002. Diabet Med.

[3] Kim SY, Dietz PM, England L, Morrow B, Callaghan WM (2007). Trends in pre-pregnancy obesity in nine states, 1993-2003. Obesity (Silver Spring).

[4] Lapolla A, Dalfrà MG, Fedele D (2008). Pregnancy complicated by type 2 diabetes: an emerging problem. Diabetes Res Clin Pract.

[5] Eidem I, Stene LC, Henriksen T, Hanssen KF, Vangen S, Vollset SE, Joner G (2010). Congenital anomalies in newborns of women with type 1 diabetes: nationwide population-based study in Norway, 1999-2004. Acta Obstet Gynecol Scand.

[6] Dabelea D, Mayer-Davis EJ, Saydah S, Imperatore G, Linder B, Divers J, Bell R, Badaru A, Talton JW, Crume T, Liese AD, Merchant AT, Lawrence JM (2014). SEARCH for Diabetes in Youth Study. Prevalence of type 1 and type 2 diabetes among children and adolescents from 2001 to 2009. JAMA.

[7] Rosenbloom AL, Joe JR, Young RS, Winter WE (1999). Emerging epidemic of type 2 diabetes in youth. Diabetes Care.

[8] Neufeld ND, Raffel LJ, Landon C, Chen YD, Vadheim CM (1998). Early presentation of type 2 diabetes in Mexican-American youth. Diabetes Care.

[9] Feig DS, Palda VA (2002). Type 2 diabetes in pregnancy: a growing concern. Lancet.

[10] Platt MJ, Stanisstreet M, Casson IF, Howard CV, Walkinshaw S, Pennycook S, McKendrick O (2002). St Vincent’s Declaration 10 years on: outcomes of diabetic pregnancies. Diabet Med.

[11] Chauhan SP, Laye MR, Lutgendorf M, McBurney JW, Keiser SD, Magann EF, Morrison JC (2014). A multicenter assessment of 1,177 cases of shoulder dystocia: lessons learned. Am J Perinatol.

[12] Bartsch E, Medcalf KE, Park AL, Ray JG, and High Risk of Pre-eclampsia Identification Group (2016). Clinical risk factors for pre-eclampsia determined in early pregnancy: systematic review and meta-analysis of large cohort studies. BMJ.

[13] Persson M, Norman M, Hanson U (2009). Obstetric and perinatal outcomes in type 1 diabetic pregnancies: A large, population-based study. Diabetes Care.

[14] Jensen DM, Damm P, Moelsted-Pedersen L, Ovesen P, Westergaard JG, Moeller M, Beck-Nielsen H (2004). Outcomes in type 1 diabetic pregnancies: a nationwide, population-based study. Diabetes Care.

[15] Evers IM, de Valk HW, Visser GH (2004). Risk of complications of pregnancy in women with type 1 diabetes: nationwide prospective study in the Netherlands. BMJ.

[16] Yang J, Cummings EA, O’connell C, Jangaard K (2006). Fetal and neonatal outcomes of diabetic pregnancies. Obstet Gynecol.

[17] Dekker GA, Lee SY, North RA, McCowan LM, Simpson NA, Roberts CT (2012). Risk factors for preterm birth in an international prospective cohort of nulliparous women. PLoS One.

[18] Mirghani H, Begam M, Bekdache G, Khan F (2012). Specialised fetal and maternal service: outcome of pre-gestational diabetes. J Obstet Gynaecol.

[19] Yu L, Cheng YJ, Cheng ML, Yao YM, Zhang Q, Zhao XK, Liu HJ, Hu YX, Mu M, Wang B, Yang GZ, Zhu LL, Zhang S (2015). Quantitative assessment of common genetic variations in HLA-DP with hepatitis B virus infection, clearance and hepatocellular carcinoma development. Sci Rep.

[20] Metzger BE, Lowe LP, Dyer AR, Trimble ER, Chaovarindr U, Coustan DR, Hadden DR, McCance DR, Hod M, McIntyre HD, Oats JJ, Persson B, Rogers MS, Sacks DA, and HAPO Study Cooperative Research Group (2008). Hyperglycemia and adverse pregnancy outcomes. N Engl J Med.

[21] Parretti E, Mecacci F, Papini M, Cioni R, Carignani L, Mignosa M, La Torre P, Mello G (2001). Third-trimester maternal glucose levels from diurnal profiles in nondiabetic pregnancies: correlation with sonographic parameters of fetal growth. Diabetes Care.

[22] Sosenko IR, Kitzmiller JL, Loo SW, Blix P, Rubenstein AH, Gabbay KH (1979). The infant of the diabetic mother: correlation of increased cord C-peptide levels with macrosomia and hypoglycemia. N Engl J Med.

[23] Evagelidou EN, Kiortsis DN, Bairaktari ET, Giapros VI, Cholevas VK, Tzallas CS, Andronikou SK (2006). Lipid profile, glucose homeostasis, blood pressure, and obesity-anthropometric markers in macrosomic offspring of nondiabetic mothers. Diabetes Care.

[24] Ornoy A (2005). Growth and neurodevelopmental outcome of children born to mothers with pregestational and gestational diabetes. Pediatr Endocrinol Rev.

[25] Zhu Y, Olsen SF, Mendola P, Yeung EH, Vaag A, Bowers K, Liu A, Bao W, Li S, Madsen C, Grunnet LG, Granström C, Hansen S (2016). Growth and obesity through the first 7 y of life in association with levels of maternal glycemia during pregnancy: a prospective cohort study. Am J Clin Nutr.

[26] Persson B, Hanson U (1996). Fetal size at birth in relation to quality of blood glucose control in pregnancies complicated by pregestational diabetes mellitus. Br J Obstet Gynaecol.

[27] Patel EM, Goodnight WH, James AH, Grotegut CA (2015). Temporal trends in maternal medical conditions and stillbirth. Am J Obstet Gynecol.

[28] Teramo K, Kari MA, Eronen M, Markkanen H, Hiilesmaa V (2004). High amniotic fluid erythropoietin levels are associated with an increased frequency of fetal and neonatal morbidity in type 1 diabetic pregnancies. Diabetologia.

[29] Lauenborg J, Mathiesen E, Ovesen P, Westergaard JG, Ekbom P, Mølsted-Pedersen L, Damm P (2003). Audit on stillbirths in women with pregestational type 1 diabetes. Diabetes Care.

[30] Rackham O, Paize F, Weindling AM (2009). Cause of death in infants of women with pregestational diabetes mellitus and the relationship with glycemic control. Postgrad Med.

[31] Innes KE, Wimsatt JH, McDuffie R (2001). Relative glucose tolerance and subsequent development of hypertension in pregnancy. Obstet Gynecol.

[32] Becker T, Vermeulen MJ, Wyatt PR, Meier C, Ray JG (2007). Prepregnancy diabetes and risk of placental vascular disease. Diabetes Care.

[33] Hanson U, Persson B (1998). Epidemiology of pregnancy-induced hypertension and preeclampsia in type 1 (insulin-dependent) diabetic pregnancies in Sweden. Acta Obstet Gynecol Scand.

[34] Vangen S, Stoltenberg C, Holan S, Moe N, Magnus P, Harris JR, Stray-Pedersen B (2003). Outcome of pregnancy among immigrant women with diabetes. Diabetes Care.

[35] Sibai BM, Caritis S, Hauth J, Lindheimer M, VanDorsten JP, MacPherson C, Klebanoff M, Landon M, Miodovnik M, Paul R, Meis P, Dombrowski M, Thurnau G, and National Institute of Child Health and Human Development Network of Maternal-Fetal Medicine Units (2000). Risks of preeclampsia and adverse neonatal outcomes among women with pregestational diabetes mellitus. Am J Obstet Gynecol.

[36] Balsells M, García-Patterson A, Gich I, Corcoy R (2009). Maternal and fetal outcome in women with type 2 versus type 1 diabetes mellitus: a systematic review and metaanalysis. J Clin Endocrinol Metab.

[37] Landon MB, Mele L, Spong CY, Carpenter MW, Ramin SM, Casey B, Wapner RJ, Varner MW, Rouse DJ, Thorp JM, Sciscione A, Catalano P, Harper M, and Eunice Kennedy Shriver National Institute of Child Health, and Human Development (NICHD) Maternal–Fetal Medicine Units (MFMU) Network (2011). The relationship between maternal glycemia and perinatal outcome. Obstet Gynecol.

[38] Klingensmith GJ, Pyle L, Nadeau KJ, Barbour LA, Goland RS, Willi SM, Linder B, White NH, and TODAY Study Group (2016). Pregnancy Outcomes in Youth With Type 2 Diabetes: The TODAY Study Experience. Diabetes Care.

[39] Yu L, Zhang BF, Cheng ML, Zhao XK, Zhang Q, Hu YX, Liu HJ, Mu M, Wang B, Yang GZ, Zhu LL, Zhang S, Yao YM (2016 May 17). Quantitative assessment of mutations in hepatitis B virus genome with liver cirrhosis and hepatocellular carcinoma development. Oncotarget.

[40] Stroup DF, Berlin JA, Morton SC, Olkin I, Williamson GD, Rennie D, Moher D, Becker BJ, Sipe TA, Thacker SB (2000). Meta-analysis of observational studies in epidemiology: a proposal for reporting. Meta-analysis Of Observational Studies in Epidemiology (MOOSE) group. JAMA.

[41] Stang A (2010). Critical evaluation of the Newcastle-Ottawa scale for the assessment of the quality of nonrandomized studies in meta-analyses. Eur J Epidemiol.

[42] Aune D, Chan DS, Greenwood DC, Vieira AR, Rosenblatt DA, Vieira R, Norat T (2012). Dietary fiber and breast cancer risk: a systematic review and meta-analysis of prospective studies. Ann Oncol.

[43] DerSimonian R, Laird N (1986). Meta-analysis in clinical trials. Control Clin Trials.

[44] Higgins JP, Thompson SG (2002). Quantifying heterogeneity in a meta-analysis. Stat Med.

[45] Egger M, Davey Smith G, Schneider M, Minder C (1997). Bias in meta-analysis detected by a simple, graphical test. BMJ.

[46] Begg CB, Mazumdar M (1994). Operating characteristics of a rank correlation test for publication bias. Biometrics.

