# Supplementary material for: Quantitative assessment of the effect of pre-gestational diabetes and risk of adverse maternal, perinatal and neonatal outcomes
Source: Oncotarget. 2017 May 11;8(37):61048–56. doi: 10.18632/oncotarget.17824 (PMC5617405; doi:10.18632/oncotarget.17824)
Supplement: Supplementary file 1 [file oncotarget-08-61048-s001.pdf]

Quantitative assessment of the effect of pre-gestational diabetes and risk of adverse maternal, perinatal and neonatal outcomes

SUPPLEMENTARY MATERIALS

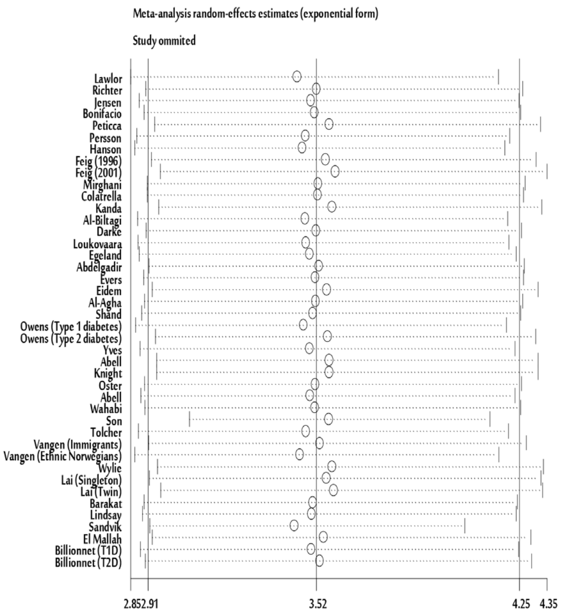

(a) Cesarean section

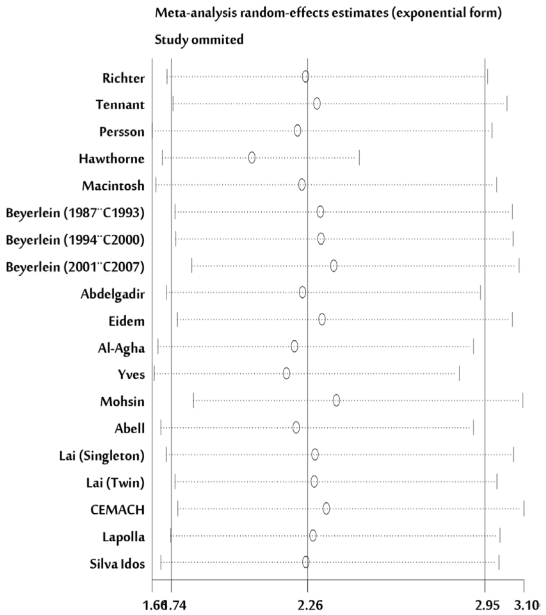

(b) Neonatal death

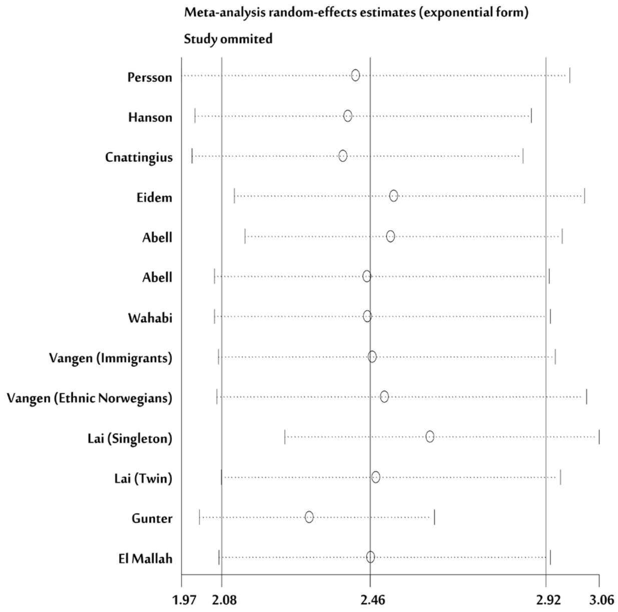

(c) Low Apgar score

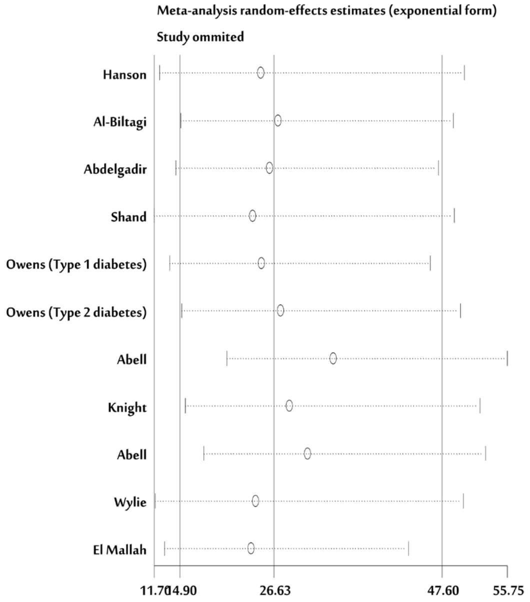

(d) Neonatal hypoglycemia

(Continued)

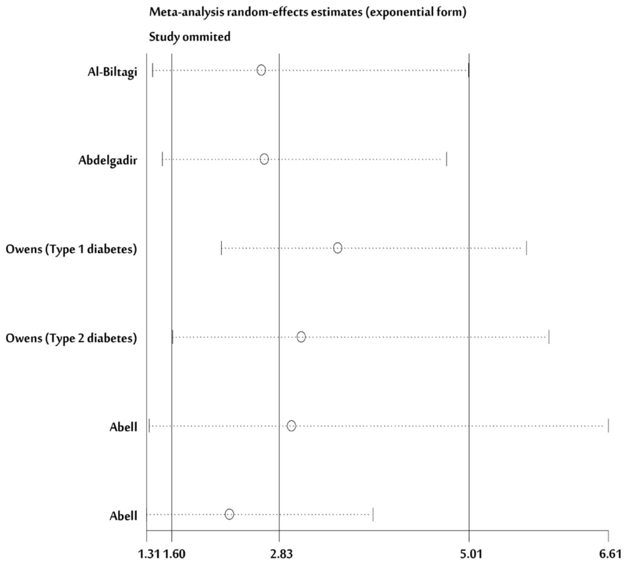

(e) Jaundice

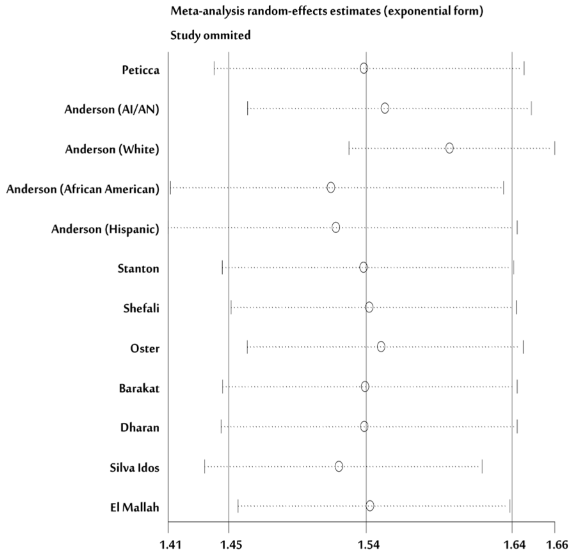

(f) Low birth weight

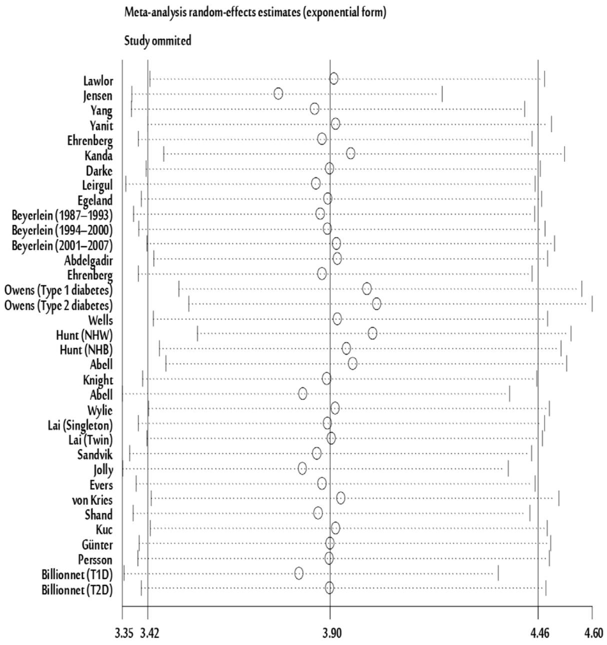

(g) Large for gestational age

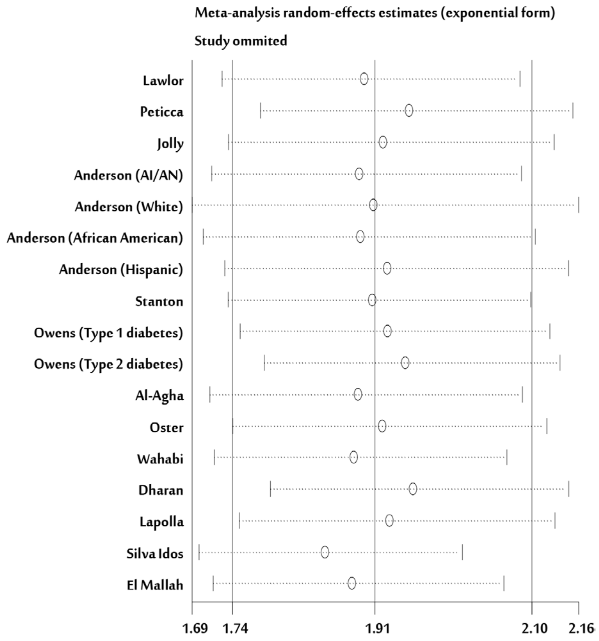

(h) Macrosomia

(Continued)

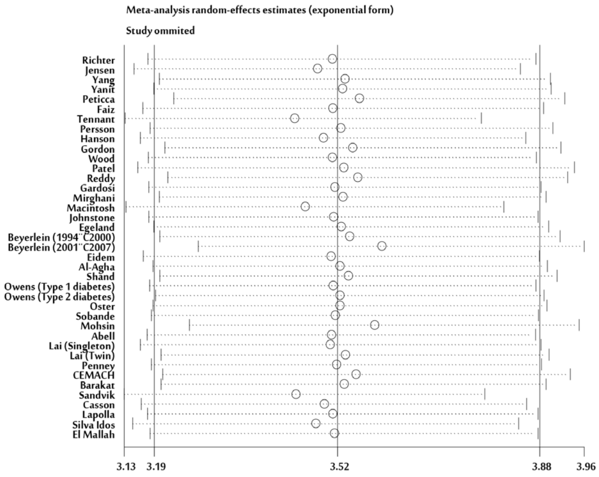

(i) Stillbirth

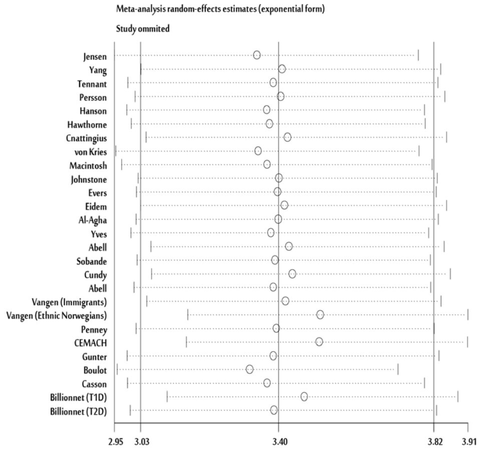

(j) Perinatal death

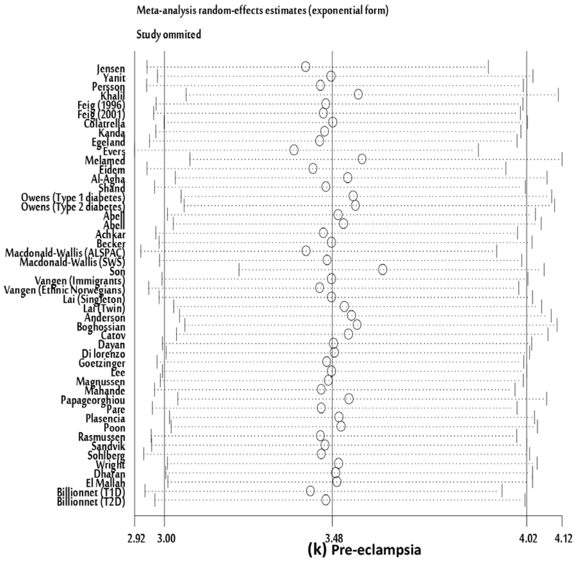

(k) Pre-eclampsia

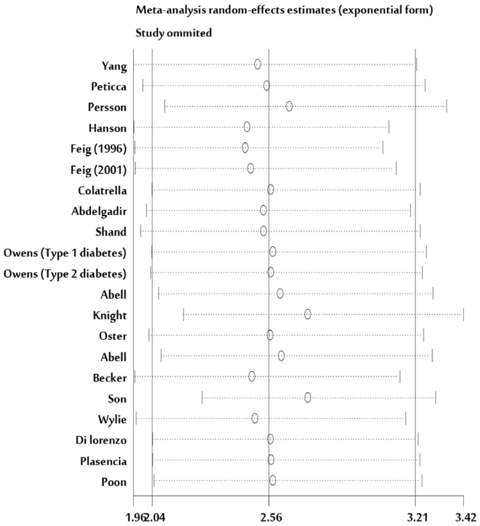

(l) Pregnancy induced hypertension

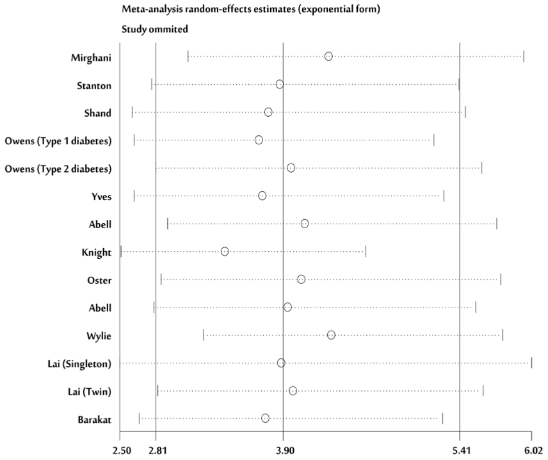

(m) NICU admission

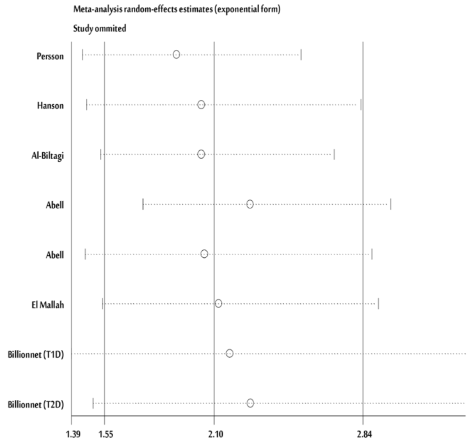

(n) Respiratory distress

(Continued)

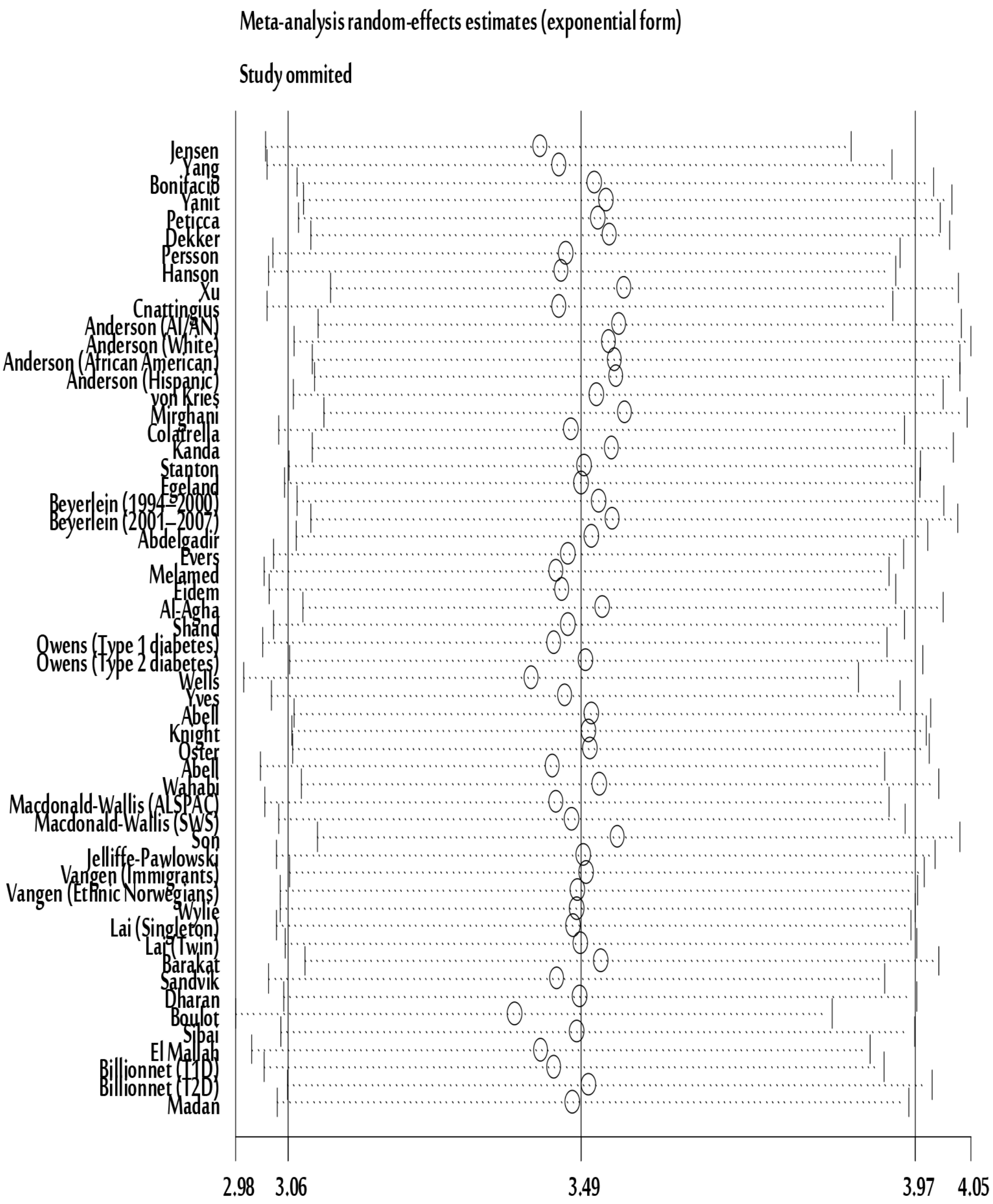

(q) Preterm delivery

Supplementary Figure 1: Result of sensitivity analyses for pre-gestational diabetes and adverse pregnancy risk.

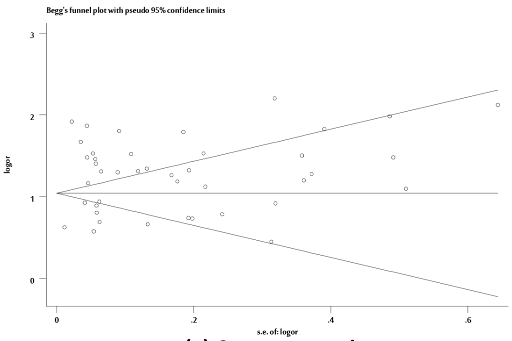

(a) Cesarean section

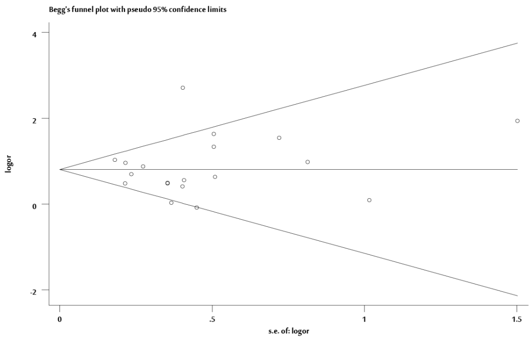

(b) Neonatal death

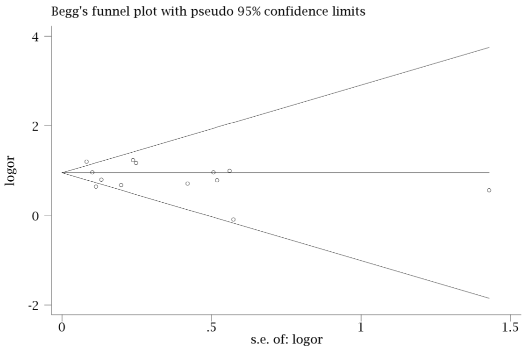

(c) Low Apgar score

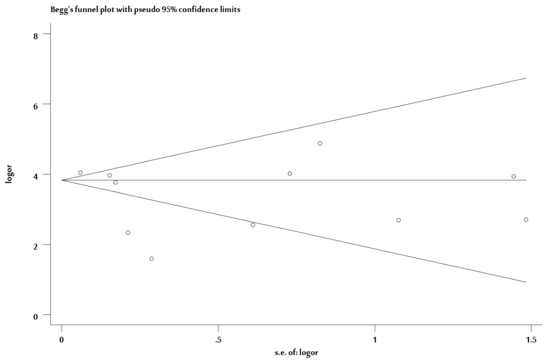

(d) Neonatal hypoglycemia

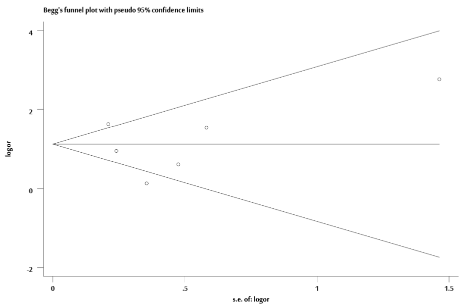

(e) Jaundice

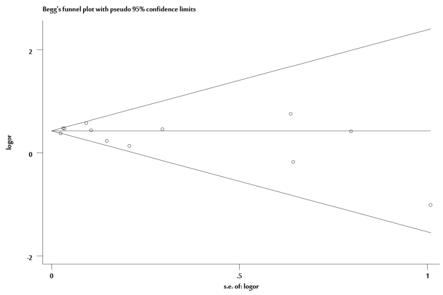

(f) Low birth weight

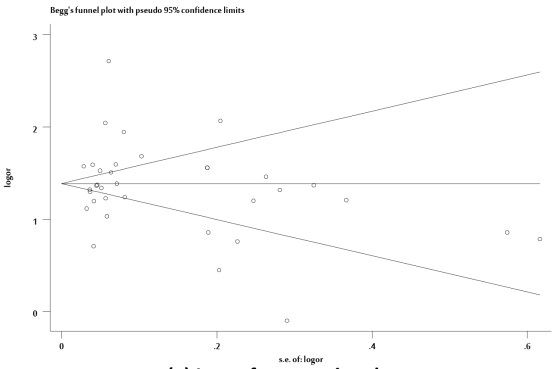

(g) Large for gestational age

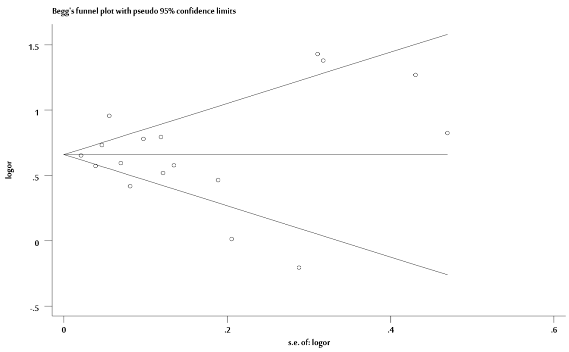

(h) Macrosomia

(Continued)

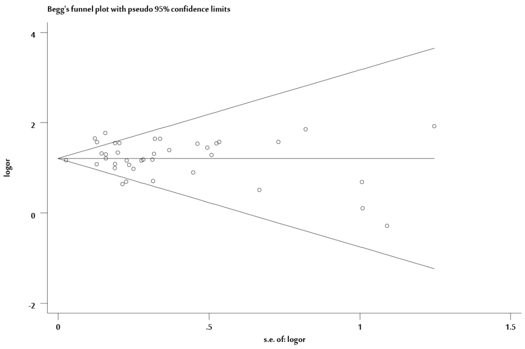

(i) Stillbirth

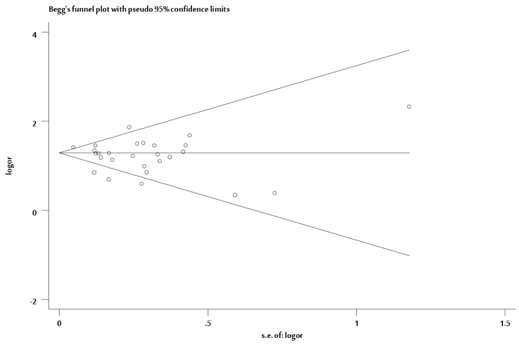

(j) Perinatal death

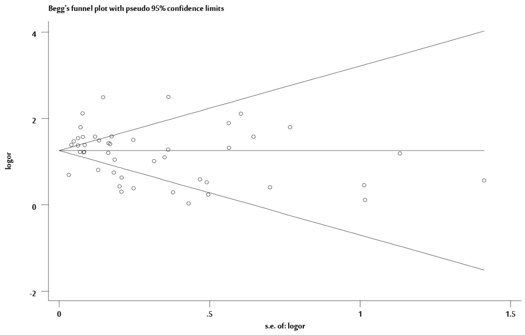

(k) Pre-eclampsia

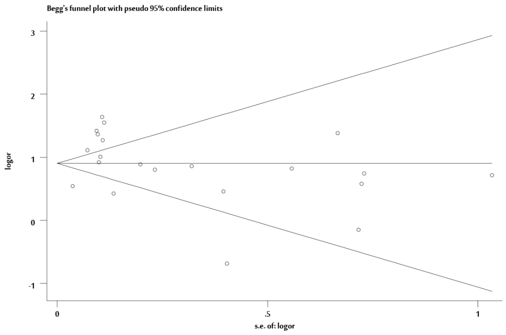

(l) Pregnancy induced hypertension

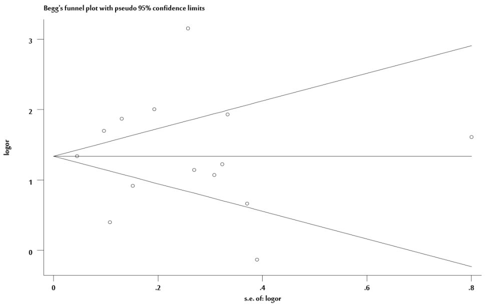

(m) NICU admission

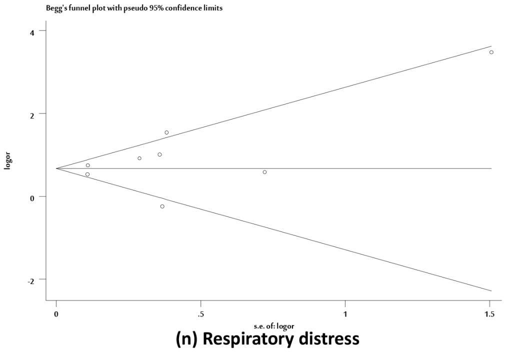

(n) Respiratory distress

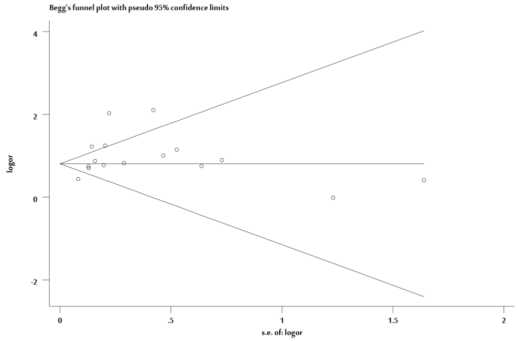

(o) Shoulder dystocia

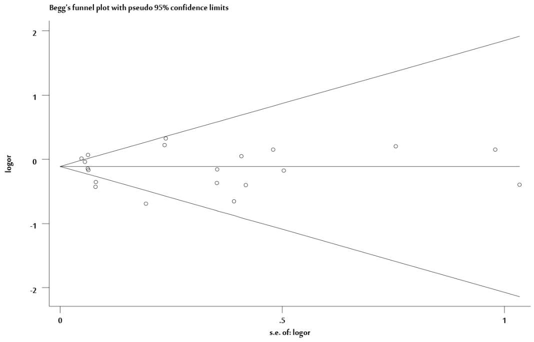

(p) Small for gestational age

(Continued)

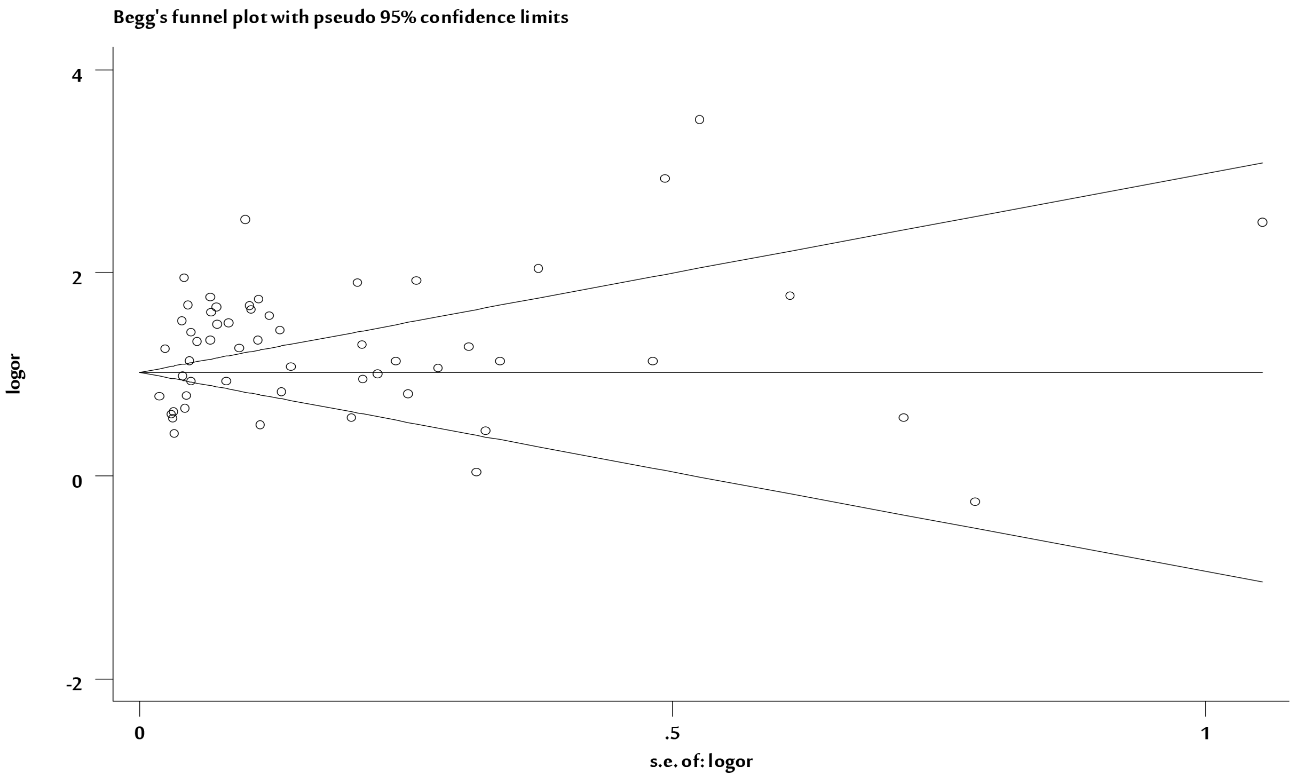

(q) Preterm delivery

Supplementary Figure 2: Begg's funnel plot for pre-gestational diabetes and adverse pregnancy risk.

Supplementary Table 1: Characteristics of the studies included in the meta-analysis

See Supplementary File 1

Supplementary Table 2: Associations of pre-gestational diabetes and adverse pregnancy outcomes stratified by sample size

Outcomes	Large study (no. PGD cases ≥ 1,000)				Medium sized study (500 ≤ no. PGD cases < 1,000)			
	No. of datasets	OR (95% CI)	P(Z)	P(Q)	No. of datasets	OR (95% CI)	P(Z)	P(Q)
Preterm delivery (< 37 weeks)	22	2.92 (2.46-3.46)	$<10^{-5}$	$<10^{-5}$	16	4.24 (3.56-5.06)	$<10^{-5}$	$<10^{-5}$
Macrosomia (> 4 kg)	6	1.87 (1.73-2.01)	$<10^{-5}$	0.005	4	2.07 (1.67-2.56)	$<10^{-5}$	0.002
LGA (> 90th percentile)	17	4.27 (3.54-5.15)	$<10^{-5}$	$<10^{-5}$	7	4.19 (3.63-4.83)	$<10^{-5}$	$<10^{-4}$
LBW (< 2500 g)	5	1.53 (1.43-1.64)	$<10^{-5}$	0.02	2	1.47 (0.96-2.26)	0.07	0.05
SGA (< 10th percentile)	8	0.87 (0.78-0.98)	0.02	$<10^{-5}$	3	0.74 (0.50-1.10)	0.14	0.01
Perinatal mortality	11	3.35 (2.90-3.86)	$<10^{-5}$	0.007	11	3.49 (2.77-4.40)	$<10^{-5}$	0.02
Neonatal death	10	1.87 (1.47-2.37)	$<10^{-5}$	0.16	5	2.36 (1.59-3.50)	$<10^{-5}$	0.18
Stillbirth	17	3.31 (2.90-3.76)	$<10^{-5}$	$<10^{-4}$	13	3.92 (3.35-4.58)	$<10^{-5}$	0.24
Pregnancy induced hypertension	7	2.84 (1.97-4.09)	$<10^{-5}$	$<10^{-5}$	4	3.41 (2.68-4.33)	$<10^{-5}$	0.003
Pre-eclampsia	16	4.03 (3.18-5.12)	$<10^{-5}$	$<10^{-5}$	14	3.10 (2.47-3.89)	$<10^{-5}$	$<10^{-5}$
Caesarean section	14	3.04 (2.31-4.00)	$<10^{-5}$	$<10^{-5}$	10	3.82 (2.82-5.18)	$<10^{-5}$	$<10^{-5}$
Shoulder dystocia	9	2.30 (1.86-2.84)	$<10^{-5}$	$<10^{-4}$	1	7.62 (4.94-11.76)	$<10^{-5}$	NA
NICU admission	3	4.19 (3.08-5.70)	$<10^{-5}$	0.002	3	2.90 (1.21-6.95)	0.02	$<10^{-5}$
5-minutes Apgar score < 7	7	2.37 (1.93-2.91)	$<10^{-5}$	0.002	2	3.32 (2.38-4.65)	$<10^{-5}$	0.86
Neonatal hypoglycemia	1	56.80 (50.55-63.83)	$<10^{-5}$	NA	2	49.04 (40.32-59.64)	$<10^{-5}$	0.35
Respiratory distress	3	2.14 (1.54-2.98)	$<10^{-4}$	0.03	1	2.74 (1.36-5.53)	0.005	NA

Supplementary Table 3: Associations of pre-gestational diabetes and adverse pregnancy outcomes stratified by geographic region

Outcomes	North American				Europe				Oceania			
	No. of datasets	OR (95% CI)	P(Z)	P(Q)	No. of datasets	OR (95% CI)	P(Z)	P(Q)	No. of datasets	OR (95% CI)	P(Z)	P(Q)
Preterm delivery (< 37 weeks)	16	3.26 (2.74-3.88)	$<10^{-5}$	$<10^{-5}$	22	4.28 (3.50-5.22)	$<10^{-5}$	$<10^{-5}$	6	3.95 (2.04-7.66)	$<10^{-4}$	$<10^{-5}$
Macrosomia (> 4 kg)	7	1.76 (1.58-1.96)	$<10^{-5}$	0.002	7	1.88 (1.52-2.34)	$<10^{-5}$	$<10^{-4}$	NA	NA	NA	NA
LGA (> 90th percentile)	10	3.64 (2.91-4.55)	$<10^{-5}$	$<10^{-5}$	19	4.14 (3.50-4.91)	$<10^{-5}$	$<10^{-5}$	4	4.07 (2.40-6.88)	$<10^{-5}$	$<10^{-4}$
LBW (<2500 g)	7	1.51 (1.41-1.61)	$<10^{-5}$	0.17	1	1.77 (1.48-2.12)	$<10^{-5}$	NA	1	0.84 (0.24-2.95)	0.78	NA
SGA (< 10th percentile)	7	0.83 (0.70-0.99)	0.04	$<10^{-4}$	7	0.82 (0.76-0.88)	$<10^{-5}$	0.54	3	0.88 (0.41-1.89)	0.74	0.09
Perinatal mortality	2	3.85 (2.56-5.79)	$<10^{-5}$	0.36	20	3.43 (3.03-3.89)	$<10^{-5}$	0.001	3	2.58 (1.52-4.39)	$<10^{-4}$	0.27
Neonatal death	2	1.94 (1.24-3.03)	0.004	0.56	14	2.38 (1.76-3.24)	$<10^{-5}$	$<10^{-4}$	2	1.93 (0.45-8.30)	0.38	0.06
Stillbirth	10	3.22 (3.07-3.38)	$<10^{-5}$	0.51	21	3.90 (3.38-4.49)	$<10^{-5}$	0.008	4	2.59 (1.99-3.38)	$<10^{-5}$	0.37
Pregnancy induced hypertension	9	3.27 (2.65-4.05)	$<10^{-5}$	$<10^{-5}$	7	1.77 (1.45-2.17)	$<10^{-5}$	0.77	3	2.00 (1.12-3.57)	0.02	0.12
Pre-eclampsia	12	3.53 (3.11-4.01)	$<10^{-5}$	0.008	25	3.99 (3.38-4.71)	$<10^{-5}$	$<10^{-5}$	4	2.62 (1.62-4.24)	$<10^{-4}$	0.003
Caesarean section	10	2.63 (2.12-3.27)	$<10^{-5}$	$<10^{-5}$	20	4.30 (3.67-5.04)	$<10^{-5}$	$<10^{-5}$	3	3.44 (2.29-5.19)	$<10^{-5}$	0.003
Shoulder dystocia	7	2.48 (1.79-3.43)	$<10^{-5}$	$<10^{-4}$	3	3.26 (2.49-4.28)	$<10^{-5}$	0.47	3	4.14 (2.35-7.29)	$<10^{-5}$	0.13
NICU admission	6	3.94 (2.19-7.09)	$<10^{-4}$	$<10^{-4}$	3	5.58 (3.68-8.47)	$<10^{-5}$	0.03	3	3.56 (1.92-6.59)	$<10^{-4}$	0.01
5-minutes Apgar score < 7	3	2.25 (1.56-3.24)	$<10^{-4}$	0.14	6	2.64 (2.19-3.19)	$<10^{-5}$	0.04	2	1.58 (0.54-4.57)	0.40	0.17
Neonatal hypoglycemia	3	42.20 (28.15-63.25)	$<10^{-5}$	0.06	2	36.17 (10.67-122.57)	$<10^{-5}$	0.31	3	14.44 (2.96-70.58)	0.001	$<10^{-5}$
Jaundice	NA	NA	NA	NA	2	1.35 (0.77-2.36)	0.29	0.41	2	3.66 (1.88-7.13)	$<10^{-4}$	0.04

Supplementary Table 4: Associations of pre-gestational diabetes and adverse pregnancy outcomes stratified by study quality

Outcomes	High quality study (≥ 7 points)				Medium quality study (5 to 6 points)			
	No. of datasets	OR (95% CI)	P(Z)	P(Q)	No. of datasets	OR (95% CI)	P(Z)	P(Q)
Preterm delivery (< 37 weeks)	27	3.31 (2.79-3.92)	$<10^{-5}$	$<10^{-5}$	17	3.84 (3.22-4.58)	$<10^{-5}$	$<10^{-5}$
Macrosomia (> 4 kg)	7	2.00 (1.79-2.24)	$<10^{-5}$	0.005	7	1.95 (1.57-2.42)	$<10^{-5}$	$<10^{-4}$
LGA (> 90th percentile)	20	4.18 (3.54-4.93)	$<10^{-5}$	$<10^{-5}$	7	4.68 (3.57-6.14)	$<10^{-5}$	$<10^{-5}$
LBW (< 2500 g)	6	1.55 (1.45-1.66)	$<10^{-5}$	0.02	3	1.22 (0.84-1.79)	0.29	0.32
SGA (< 10th percentile)	9	0.83 (0.73-0.94)	0.004	$<10^{-4}$	5	1.05 (0.73-1.52)	0.80	0.34
Perinatal mortality	18	3.41 (2.98-3.91)	$<10^{-5}$	$<10^{-4}$	7	3.20 (2.49-4.12)	$<10^{-5}$	0.40
Neonatal death	12	1.90 (1.57-2.31)	$<10^{-5}$	0.21	6	4.66 (2.36-9.19)	$<10^{-5}$	0.04
Stillbirth	26	3.52 (3.14-3.94)	$<10^{-5}$	$<10^{-4}$	8	3.38 (2.62-4.37)	$<10^{-5}$	0.88
Pregnancy induced hypertension	8	3.07 (2.18-4.31)	$<10^{-5}$	$<10^{-5}$	7	2.66 (2.11-3.35)	$<10^{-5}$	0.11
Pre-eclampsia	23	3.95 (3.28-4.75)	$<10^{-5}$	$<10^{-5}$	17	2.82 (2.17-3.68)	$<10^{-5}$	$<10^{-5}$
Caesarean section	18	3.53 (2.64-4.72)	$<10^{-5}$	$<10^{-5}$	12	3.38 (2.77-4.14)	$<10^{-5}$	$<10^{-5}$
Shoulder dystocia	8	2.18 (1.77-2.70)	$<10^{-5}$	0.001	4	5.02 (2.94-8.56)	$<10^{-5}$	0.02
NICU admission	2	3.79 (3.47-4.13)	$<10^{-5}$	0.38	7	2.70 (1.60-4.53)	$<10^{-4}$	$<10^{-5}$
5-minutes Apgar score < 7	8	2.44 (2.01-2.95)	$<10^{-5}$	0.003	5	2.57 (1.63-4.04)	$<10^{-4}$	0.32
Neonatal hypoglycemia	1	43.06 (30.72-60.34)	$<10^{-5}$	NA	8	25.59 (11.39-57.50)	$<10^{-5}$	$<10^{-5}$
Respiratory distress	4	2.20 (1.64-2.95)	$<10^{-5}$	0.04	3	1.51 (0.65-3.49)	0.34	0.05
Jaundice	NA	NA	NA	NA	2	2.66 (1.88-7.13)	$<10^{-4}$	0.04

Supplementary Table 5: Associations of pre-gestational diabetes and adverse pregnancy outcomes stratified by adjustment

Outcomes	Adjusted study				Unadjusted study			
	No. of datasets	OR (95% CI)	P(Z)	P(Q)	No. of datasets	OR (95% CI)	P(Z)	P(Q)
Preterm delivery (< 37 weeks)	23	3.00 (2.55-3.52)	$<10^{-5}$	$<10^{-5}$	32	3.98 (3.26-4.86)	$<10^{-5}$	$<10^{-5}$
Macrosomia (> 4 kg)	7	1.92 (1.74-2.12)	$<10^{-5}$	0.001	10	1.82 (1.48-2.22)	$<10^{-5}$	$<10^{-4}$
LGA (> 90th percentile)	12	4.26 (3.47-5.23)	$<10^{-5}$	$<10^{-5}$	23	3.71 (3.12-4.42)	$<10^{-5}$	$<10^{-5}$
LBW (< 2500 g)	5	1.53 (1.43-1.64)	$<10^{-5}$	0.02	7	1.50 (1.19-1.90)	0.001	0.29
SGA (< 10th percentile)	6	0.75 (0.57-1.01)	0.05	$<10^{-4}$	14	0.91 (0.82-1.01)	0.07	0.01
Perinatal mortality	12	2.98 (2.46-3.62)	$<10^{-5}$	$<10^{-4}$	15	3.77 (3.38-4.21)	$<10^{-5}$	0.58
Neonatal death	8	1.92 (1.45-2.56)	$<10^{-5}$	0.18	11	2.62 (1.72-4.00)	$<10^{-5}$	$<10^{-4}$
Stillbirth	15	3.14 (2.82-3.50)	$<10^{-5}$	0.46	24	3.84 (3.31-4.46)	$<10^{-5}$	$<10^{-5}$
Pregnancy induced hypertension	8	2.48 (1.74-3.53)	$<10^{-5}$	$<10^{-5}$	13	2.74 (2.16-3.47)	$<10^{-5}$	$<10^{-4}$
Pre-eclampsia	23	3.39 (2.78-4.13)	$<10^{-5}$	$<10^{-5}$	25	3.61 (2.93-4.43)	$<10^{-5}$	$<10^{-5}$
Caesarean section	17	3.14 (2.43-4.07)	$<10^{-5}$	$<10^{-5}$	25	3.80 (3.18-4.54)	$<10^{-5}$	$<10^{-5}$
Shoulder dystocia	10	2.37 (1.89-2.99)	$<10^{-5}$	$<10^{-4}$	6	3.80 (2.21-6.53)	$<10^{-4}$	0.05
NICU admission	4	3.41 (2.69-4.32)	$<10^{-5}$	0.25	10	4.32 (2.65-7.05)	$<10^{-5}$	$<10^{-5}$
5-minutes Apgar score < 7	9	2.20 (1.96-2.47)	$<10^{-5}$	0.47	4	3.30 (2.85-3.81)	$<10^{-5}$	0.97
Neonatal hypoglycemia	2	7.29 (3.53-15.07)	$<10^{-5}$	0.04	9	50.16 (41.21-61.06)	$<10^{-5}$	0.18
Respiratory distress	5	1.97 (1.42-2.73)	$<10^{-4}$	0.006	3	3.04 (1.17-7.94)	0.02	0.22
Jaundice	2	3.66 (1.88-7.13)	$<10^{-4}$	0.04	4	2.28 (1.00-5.22)	0.05	0.09

Supplementary Table 6: P-value of covariates in meta-regression analysis

Outcomes	Study design	Geographic region	Sample size	Term	Adjustment	Study quality	Publication year
Preterm delivery (< 37 weeks)	0.43	**0.002**	0.06	0.98	**0.03**	0.55	0.09
Shoulder dystocia	0.71	0.95	0.07	0.61	**0.04**	0.06	0.14
Macrosomia (> 4 kg)	0.94	0.82	0.93	0.79	0.42	0.37	0.17
LGA (> 90th percentile)	0.99	0.31	0.12	0.47	0.64	**0.04**	0.06
LBW (< 2500 g)	0.27	0.50	0.86	0.55	0.69	0.33	0.40
SGA (< 10th percentile)	0.59	0.20	0.97	0.99	0.17	0.40	0.60
Perinatal mortality	0.56	0.43	0.84	0.92	0.09	0.71	0.49
Neonatal death	0.94	0.90	**0.001**	0.80	0.28	**0.001**	0.05
Stillbirth	**$<10^{-4}$**	**$<10^{-4}$**	0.17	0.69	**0.03**	0.78	0.29
NICU admission	0.84	0.40	0.75	0.95	0.44	0.13	0.88
Pregnancy induced hypertension	0.32	0.91	0.08	0.76	0.86	0.20	0.10
Pre-eclampsia	0.56	0.67	**0.01**	0.46	0.59	0.29	0.07
Caesarean section	0.99	0.07	0.14	0.87	0.11	0.84	0.18
5-minutes Apgar score < 7	0.45	0.16	0.84	0.93	**$<10^{-4}$**	0.36	0.05
Neonatal hypoglycemia	**$<10^{-5}$**	0.58	0.05	0.65	**$<10^{-5}$**	0.55	0.65
Respiratory distress	0.56	0.86	0.13	0.88	0.56	0.59	0.81
Jaundice	0.29	**0.02**	NA	0.91	0.29	0.94	0.37

Supplementary Table 7: P-value of publication bias analysis

Outcomes	Egger's test	Begg's test
Preterm delivery (< 37 weeks)	0.21	0.48
Shoulder dystocia	0.12	0.79
Macrosomia (>4 kg)	0.97	0.93
LGA (> 90th percentile)	0.89	0.91
LBW (< 2500 g)	0.56	0.27
SGA (< 10th percentile)	0.52	0.95
Perinatal mortality	0.15	0.88
Neonatal death	0.77	0.73
Stillbirth	0.43	0.65
NICU admission	0.90	0.48
Pregnancy induced hypertension	0.42	0.25
Pre-eclampsia	0.78	0.53
Caesarean section	0.10	0.12
5-minutes Apgar score < 7	0.34	0.63
Neonatal hypoglycemia	0.16	0.59
Respiratory distress	0.38	0.22
Jaundice	0.86	0.85
