# Supplementary material for: Quantitative assessment of the effect of pre-gestational diabetes and risk of adverse maternal, perinatal and neonatal outcomes
Source: Oncotarget. 2017 May 11;8(37):61048–56. doi: 10.18632/oncotarget.17824 (PMC5617405; doi:10.18632/oncotarget.17824)
Supplement: Supplementary file 2 [file oncotarget-08-61048-s002.docx]

**Supplementary Table 1** Characteristics of the studies included in the meta-analysis

| **Study** | **Year** | **Study design** | **Pregnancy outcomes** | **Cases/Controls** | **Type of diabetes** | **Country** | **Term** | **Quality score** |
| --- | --- | --- | --- | --- | --- | --- | --- | --- |
| Lawlor [1] | 2010 | Prospective cohort | Macrosomia, LGA, CS | 40/10,126 | PGD | UK | singleton | 7 |
| Richter [2] | 2007 | Prospective cohort | Stillbirth, CS, Neonatal death | 163/62,535 | PGD | Germany | singleton | 6 |
| Jensen [3] | 2004 | Nationwide prospective multicenter | Preterm delivery, LGA, Stillbirth, Perinatal mortality, PE, CS | 1,215/70,089 | T1D | Denmark | 97.7% singleton | 8 |
| Yang [4] | 2006 | Population-based cohort | Preterm birth, LGA, SGA, Stillbirth, Perinatal mortality, PIH | 516/150,589 | PGD | Canada | singleton | 8 |
| Bonifacio [5] | 2008 | Prospective cohort | Preterm delivery, CS | 904/556 | T1D | Germany | singleton | 5 |
| Yanit [6] | 2012 | Retrospective cohort | Preterm birth, LGA, SGA, Shoulder dystocia, PE, IUFD | 3,718/522,377 | PGD | USA | singleton | 8 |
| Peticca [7] | 2009 | Retrospective population-based multi-cohort | Preterm birth, Shoulder dystocia, Macrosomia, Stillbirth, LBW, PE, CS | 1,420/115,996 | T1D, T2D | Canada | 96.5% singleton | 8 |
| Ehrenberg [8] | 2004 | Retrospective cohort | LGA | 133/12,420 | PGD | USA | singleton | 5 |
| Faiz [9] | 2012 | Retrospective case-control | Stillbirths | 2,784/308,748 | PGD | USA | singleton | 7 |
| Dekker [10] | 2012 | Prospective international multicentre cohort | Preterm birth | 146/2,903 | T2D | Australia | singleton | 4 |
| Tennant [11] | 2014 | Population-based cohort | Stillbirth, Perinatal mortality, Neonatal death | 1,548/395,844 | PGD | UK | singleton | 8 |
| Persson [12] | 2009 | Prospective population-based cohort | Stillbirth, Perinatal mortality, Preterm birth, LGA, SGA, PE, CS, PIH, Neonatal mortality, Apgar score, Respiratory distress | 5,089/1,260,207 | T1D | Sweden | singleton | 9 |
| Hanson [13] | 1993 | Prospective nationwide | Preterm birth, Perinatal death, Stillbirths, LGA, SGA, Hypoglycaemia, CS, PIH, Apgar score, Respiratory distress | 491/279,000 | T1D | USA | unknown | 7 |
| Gordon [14] | 2013 | Prospective cohort | Stillbirths | 1,906/326,911 | PGD | Australia | singleton | 8 |
| Xu [15] | 2014 | Population-based retrospective | Preterm births | 2,447/372,954 | PGD | Australia | singleton | 8 |
| Wood [16] | 2003 | Retrospective cohort and nested case-control | Stillbirths | 416/12,727 | PGD | UK | unknown | 7 |
| Hawthorne [17] | 1997 | Prospective population based | Perinatal mortality, Neonatal mortality | 111/37,409 | PGD | UK | singleton | 5 |
| Khalil [18] | 2013 | Retrospective cohort | LGA, SGA, PE | 545/75,613 | T1D, T2D | UK | singleton | 6 |
| Patel [19] | 2015 | Retrospective case-control | Stillbirths | 130,970/12,393,149 | PGD | USA | mixed | 8 |
| Jolly [20] | 2003 | Retrospective case-control | Macrosomia | 1,172/295,192 | PGD | UK | singleton | 6 |
| Cnattingius [21] | 1994 | Retrospective case-control | Preterm birth, LGA, SGA, Perinatal mortality, Apgar score | 914/4,000 | PGD | Sweden | singleton | 5 |
| Reddy [22] | 2010 | Retrospective cohort | Stillbirth | 2,633/172,176 | PGD | USA | singleton | 8 |
| Gardosi [23] | 2013 | Prospective cohort | Stillbirth | 737/90,570 | PGD | UK | singleton | 7 |
| Anderson [24] | 2016 | Population-based retrospective | Preterm delivery, Macrosomia, LBW | 32,690/4,963,260 | PGD | USA | singleton | 7 |
| von Kries [25] | 1997 | Retrospective cohort | Perinatal deaths, Macrosomia, Prematurity | 2,402/595,393 | PGD | Germany | unknown | 7 |
| Feig [26] | 2006 | Population-based retrospective cohort | Shoulder dystocia, PE, CS, PIH | 2,654/259,407 | PGD | Canada | mixed | 7 |
| Mirghani [27] | 2012 | Prospective cohort | Preterm delivery, Stillbirth, NICU, CS | 138/12,832 | PGD | United Arab Emirates | singleton | 5 |
| Macintosh [28] | 2006 | Prospective cohort | Stillbirths, Perinatal death, Neonatal death | 2,356/620,841 | T1D, T2D | UK | mixed | 8 |
| Colatrella [29] | 2009 | Retrospective case-control | Preterm birth, PE, CS, PIH | 76/60 | T2D | Italy | singleton | 2 |
| Kanda [30] | 2012 | Retrospective case-control | Preterm delivery, LGA, SGA, PE, CS | 336/1,098 | T1D, T2D | Japan | singleton | 4 |
| Al-Biltagi [31] | 2015 | Retrospective case-control | Hypoglycemia, CS, Jaundice, Respiratory distress | 20/45 | PGD | Egypt | singleton | 2 |
| Johnstone [32] | 1990 | Prospective case-control | Stillbirths, Perinatal death | 161/23,567 | PGD | UK | singleton | 4 |
| Stanton [33] | 2005 | Retrospective case-control | Preterm delivery, NICU, LBW, Macrosomia | 73/73 | PGD | USA | singleton | 2 |
| Darke [34] | 2016 | Prospective case-control | LGA, CS | 27/6,407 | PGD | UK | twin | 3 |
| Loukovaara [35] | 2004 | Retrospective case-control | Macrosomia, CS, Apgar score | 67/62 | T1D | Finland | singleton | 2 |
| Leirgul [36] | 2016 | Prospective cohort | LGA, SGA | 5,396/862,429 | PGD | Norway | singleton | 9 |
| Egeland [37] | 2002 | Prospective cohort | Stillbirths, Preterm birth, LGA, Macrosomia, Neonatal death, PE, CS | 493/140,267 | PGD | Norway | singleton | 7 |
| Beyerlein [38] | 2010 | Population-based retrospective | Stillbirths, Preterm delivery, LGA, Early neonatal death | 7,801/2,284,252 | PGD | Germany | unknown | 8 |
| Shefali [39] | 2006 | Case-control | LBW, Large babies | 79/30 | PGD | New Zealand | unknown | 1 |
| Abdelgadir [40] | 2003 | Retrospective case-control | Prematurity, LGA, SGA, Hypoglycaemia, Neonatal death, CS, PIH, Jaundice | 69/50 | T1D, T2D | Sudan | unknown | 2 |
| Evers [41] | 2004 | Prospective cohort | Prematurity, Macrosomia, Perinatal mortality, PE, CS | 323/200,679 | T1D | Netherlands | mixed | 7 |
| Ehrenberg [42] | 2004 | Retrospective case-control | LGA | 133/12,420 | PGD | USA | singleton | 5 |
| Melamed [43] | 2008 | Retrospective cohort | Preterm deliveries, PE | 448/1,038 | PGD | Israel | singleton | 5 |
| Eidem [44] | 2011 | Retrospective cohort | Stillbirth, Perinatal death, Neonatal death, Preterm birth, PE, CS, Apgar score | 1,307/1,161,092 | T1D | Norway | unknown | 8 |
| Al-Agha [45] | 2012 | Retrospective case-control | Stillbirth, Perinatal death, Neonatal death, PE, Preterm delivery, CS, Macrosomia | 600/142,498 | T1D | Ireland | unknown | 5 |
| Shand [46] | 2008 | Cross-sectional | Preterm delivery, Shoulder dystocia, Stillborn, NICU, Hypoglycaemia, PE, CS, PIH, Macrosomia, Apgar score | 1248/352,673 | PGD | Australia | singleton | 6 |
| Owens [47] | 2015 | Retrospective case-control | Preterm deliveries, Stillbirth, Shoulder dystocia, LGA, SGA, NICU, Macrosomia, Hypoglycemia, PE, PIH, CS, Jaundice | 323/660 | T1D, T2D | Ireland | singleton | 3 |
| Wells [48] | 2015 | Retrospective cohort | Preterm birth, LGA, SGA | 18/1,282 | T2D | Australia | singleton | 3 |
| Hunt [49] | 2012 | Retrospective cohort | LGA, SGA | 4,767/198,853 | PGD | USA | singleton | 8 |
| Kuc [50] | 2011 | Retrospective nested case-control | Macrosomia | 178/186 | PGD | Netherlands | singleton | 4 |
| Yves [51] | 2010 | Retrospective case-control | Perinatal death, Neonatal death, NICU, Preterm birth, CS | 361/180,842 | T1D | Belgium | unknown | 6 |
| Abell [52] | 2016 | Retrospective cohort | Preterm birth, Shoulder dystocia, Perinatal death, NICU, Hypoglycaemia, LGA, SGA, PE, CS, PIH, Apgar score, Jaundice, Respiratory distress | 138/27,075 | T2D | Australia | singleton | 5 |
| Knight [53] | 2012 | Retrospective cohort | Preterm delivery, LGA, SGA, Hypoglycemia, NICU, CS, PIH, Shoulder dystocia | 213/213 | T2D | USA | unknown | 3 |
| Oster [54] | 2014 | Retrospective case-control | Preterm, Stillbirth, NICU, LBW, High birth weight, PIH, CS | 289/26,793 | PGD | Canada | mixed | 5 |
| Sobande [55] | 2000 | Retrospective case-control | Perinatal death, Stillbirth | 26/83 | PGD | Kingdom of Saudi Arabia | singleton | 1 |
| Mohsin [56] | 2006 | Retrospective case-control | Stillbirth, Neonatal death | 2,009/434,146 | PGD | Australia | mixed | 8 |
| Cundy [57] | 2000 | Retrospective case-control | Perinatal death | 416/82,025 | T1D, T2D | New Zealand | unknown | 5 |
| Abell [58] | 2016 | Retrospective cohort | Preterm birth, Shoulder dystocia, Stillbirths, Perinatal death, NICU, Hypoglycaemia, LGA, SGA, PE, CS, PIH, Neonatal death, Apgar score, Jaundice, Respiratory distress | 107/27,075 | T1D | Australia | singleton | 5 |
| Achkar [59] | 2015 | Retrospective nested case-control | PE | 14/2,121 | PGD | Canada | singleton | 3 |
| Becker [60] | 2007 | Retrospective population-based | PE, PIH | 1,717/384,606 | PGD | Canada | singleton | 8 |
| Wahabi [61] | 2012 | Retrospective cohort | Preterm delivery, Macrosomia, CS, Apgar score | 116/2,472 | T1D, T2D | Kingdom of Saudi Arabia | singleton | 5 |
| Macdonald-Wallis [62] | 2015 | Prospective cohort | Preterm birth, SGA, PE | 55/14,272 | PGD | Kingdom of Saudi Arabia | singleton | 5 |
| Son [63] | 2015 | Retrospective population-based | Preterm delivery, PIH, PE, Shoulder dystocia, CS | 32,207/1,171,575 | PGD | Korea | mixed | 8 |
| Jelliffe-Pawlowski [64] | 2015 | Retrospective Population-based cohort | Preterm birth | 11,733/825,056 | PGD | USA | singleton | 8 |
| Tolcher [65] | 2015 | Retrospective cohort | Cesarean delivery | 16/700 | PGD | USA | singleton | 3 |
| Vangen [66] | 2003 | Retrospective case-control | Preterm birth, PE, Shoulder dystocia, CS, Perinatal deaths, Macrosomia, LBW, Apgar score | 2,266/610,787 | PGD | Norway | unknown | 8 |
| Wylie [67] | 2002 | Retrospective case-control | Preterm delivery, PIH, CS, LGA, Dystocia, Hypoglycemia, NICU | 247/71,636 | T1D | USA | mixed | 5 |
| Lai [68] | 2016 | Retrospective population-based | Preterm birth, PE, Shoulder dystocia, CS, Stillbirth, SGA, LGA, NICU, Neonatal deaths, Macrosomia, Apgar score | 2,535/311,673 | PGD | Canada | singleton, twin | 8 |
| Penney [69] | 2003 | Prospective population-based cohort | Stillbirth, Perinatal mortality | 273/55,433 | T1D | Scotland | 98.6% singleton | 8 |
| CEMACH [70] | 2005 | Retrospective population-based | Stillbirth, Perinatal death, Neonatal death | 3,808/620,841 | PGD | UK | mixed | 8 |
| Barakat [71] | 2010 | Retrospective case-control | Preterm birth, Shoulder dystocia, Stillbirth, NICU, LBW, High birth weight, CS | 54/245 | PGD | Oman | singleton | 2 |
| Lindsay [72] | 2003 | Retrospective case-control | CS | 140/49 | T1D | UK | singleton | 2 |
| Anderson [73] | 2012 | Retrospective cohort | PE | 349/18,622 | T1D, T2D | New Zealand | singleton | 7 |
| Boghossian [74] | 2014 | Longitudinal retrospective | PE | 167/26,446 | PGD | USA | singleton | 6 |
| Catov [75] | 2007 | Nationwide longitudinal prospective cohort | PE | 265/70,659 | PGD | Denmark | singleton | 7 |
| Dayan [76] | 2015 | Retrospective cohort | PE | 208/9,805 | PGD | Canada | singleton | 7 |
| Di lorenzo [77] | 2012 | Prospective cohort | PE, PIH | 23/2,095 | PGD | Italy | singleton | 4 |
| Goetzinger [78] | 2010 | Retrospective cohort | PE | 94/3,622 | PGD | USA | singleton | 5 |
| Lee [79] | 2000 | Retrospective cohort | PE | 58/29,677 | PGD | China | unknown | 5 |
| Magnussen [80] | 2007 | Prospective cohort | PE | 11/3,483 | PGD | Norway | singleton | 4 |
| Mahande [81] | 2013 | Prospective cohort | PE | 7/3,902 | PGD | Tanzania | singleton | 2 |
| Papageorghiou [82] | 2005 | Prospective cohort | PE | 145/16,661 | PGD | UK | singleton | 7 |
| Pare [83] | 2014 | Prospective cohort | PE | 57/2,580 | PGD | USA | mixed | 5 |
| Plasencia [84] | 2007 | Prospective cohort | PE, PIH, SGA | 55/5,746 | PGD | UK | singleton | 5 |
| Poon [85] | 2010 | Prospective cohort | PE, PIH | 68/8,298 | PGD | UK | singleton | 6 |
| Rasmussen [86] | 2000 | Nationwide prospective cohort | PE | 897/375,531 | PGD | Norway | singleton | 8 |
| Sandvik [87] | 2010 | Nationwide retrospective cohort | PE, Preterm birth, SGA, LGA, Stillbirth, CS | 2,204/634,499 | PGD | Norway | singleton | 8 |
| Sohlberg [88] | 2012 | Nationwide prospective cohort | PE | 2,667/497,651 | PGD | Sweden | singleton | 8 |
| Wright [89] | 2012 | Prospective cohort | PE | 411/58,473 | PGD | UK | singleton | 8 |
| Dharan [90] | 2010 | Retrospective cohort | PE, Preterm birth, LBW, Macrosomia | 127/ 12,968 | PGD | USA | mixed | 5 |
| Günter [91] | 2006 | Retrospective population-based | Preterm birth, Macrosomia, Apgar score, Perinatal mortality, SGA, LGA | 3439/905,765 | PGD | German | mixed | 7 |
| Persson [92] | 2011 | Prospective population-based cohort | LGA | 3,705/883,163 | T1D | Sweden | singleton | 8 |
| Boulot [93] | 2003 | Cross-sectional | Perinatal mortality, Preterm delivery | 435/291,126 | T1D, T2D | French | singleton | 7 |
| Casson [94] | 1997 | Population cohort | Stillbirth, Perinatal mortality | 355/2,400,000 | T1D | north west England | mixed | 8 |
| Lapolla [95] | 2008 | Prospective cohort | Stillbirth, Macrosomia, Neonatal mortality | 668/Unknown | T1D, T2D | Italy | singleton | 6 |
| Sibai [96] | 2000 | Retrospective case-control | Preterm delivery | 461/2,738 | PGD | USA | singleton | 6 |
| Silva Idos [97] | 2005 | Population-based cohort | Macrosomia, LBW, Stillbirth, Neonatal death | 706/29,234 | PGD | UK | singleton | 7 |
| El Mallah [98] | 1997 | Retrospective case-control | PE, CS, Stillbirths, Hypoglycemia, LBW, Apgar score, Macrosomia, Preterm delivery, Respiratory distress | 71/8,904 | PGD | Saudi Arabia | mixed | 5 |
| Billionnet [99] | 2017 | Cross-sectional | Preterm delivery, PE, CS, Perinatal death, Macrosomia, Respiratory distress | 3,198/735,519 | T1D, T2D | France | mixed | 7 |
| Madan [100] | 2010 | Retrospective case-control | Preterm delivery | 352/57,760 | PGD | USA | singleton | 4 |

PGD: pre-gestational diabetes; T1D: type 1 diabetes; T2D: type 2 diabetes; PE: pre-eclampsia; LGA: large-for-gestational age; SGA: small-for-gestational age; LBW: Low birth weight; NICU: neonatal intensive care unit; CS: cesarean section; PIH: pregnancy induced hypertension

**Supplementary References**

1. Lawlor DA, Fraser A, Lindsay RS et al (2010) Association of existing diabetes, gestational diabetes and glycosuria in pregnancy with macrosomia and offspring body mass index, waist and fat mass in later childhood: findings from a prospective pregnancy cohort. Diabetologia 53:89-97

2. Richter R, Bergmann RL, Dudenhausen JW (2007) Previous caesarean or vaginal delivery: which mode is a greater risk of perinatal death at the second delivery? Eur J Obstet Gynecol Reprod Biol 132:51-57

3. Jensen DM, Damm P, Moelsted-Pedersen L et al (2004) Outcomes in type 1 diabetic pregnancies: a nationwide, population-based study. Diabetes Care 27:2819-2823

4. Yang J, Cummings EA, O'connell C (2006) Fetal and neonatal outcomes of diabetic pregnancies. Obstet Gynecol 108:644-650

5. Bonifacio E, Pflüger M, Marienfeld S et al (2008) Maternal type 1 diabetes reduces the risk of islet autoantibodies: relationships with birthweight and maternal HbA(1c). Diabetologia 51:1245-1252

6. Yanit KE, Snowden JM, Cheng YW et al (2012) The impact of chronic hypertension and pregestational diabetes on pregnancy outcomes. Am J Obstet Gynecol 207:333.e1-6

7. Peticca P, Keely EJ, Walker MC et al (2009) Pregnancy outcomes in diabetes subtypes: how do they compare? A province-based study of Ontario, 2005-2006. J Obstet Gynaecol Can 31:487-496

8. Ehrenberg HM, Mercer BM, Catalano PM (2004) The influence of obesity and diabetes on the prevalence of macrosomia. Am J Obstet Gynecol 191:964-968

9. Faiz AS, Demissie K, Rich DQ et al (2012) Trends and risk factors of stillbirth in New Jersey 1997-2005. J Matern Fetal Neonatal Med 25:699-705

10. Dekker GA, Lee SY, North RA et al (2012) Risk factors for preterm birth in an international prospective cohort of nulliparous women. PLoS One 7:e39154

11. Tennant PW, Glinianaia SV, Bilous RW et al (2014) Pre-existing diabetes, maternal glycated haemoglobin, and the risks of fetal and infant death: a population-based study. Diabetologia 57:285-294

12. Persson M, Norman M, Hanson U (2009) Obstetric and perinatal outcomes in type 1 diabetic pregnancies: A large, population-based study. Diabetes Care 32:2005-2009

13. Hanson U, Persson B (1993) Outcome of pregnancies complicated by type 1 insulin-dependent diabetes in Sweden: acute pregnancy complications, neonatal mortality and morbidity. Am J Perinatol 10:330-333

14. Gordon A, Raynes-Greenow C, McGeechan K et al (2013) Risk factors for antepartum stillbirth and the influence of maternal age in New South Wales Australia: a population based study. BMC Pregnancy Childbirth 13:12

15. Xu XK, Wang YA, Li Z et al (2014) Risk factors associated with preterm birth among singletons following assisted reproductive technology in Australia 2007-2009--a population-based retrospective study. BMC Pregnancy Childbirth 14:406

16. Wood SL, Jick H, Sauve R (2003) The risk of stillbirth in pregnancies before and after the onset of diabetes. Diabet Med 20:703-707

17. Hawthorne G, Robson S, Ryall EA et al (1997) Prospective population based survey of outcome of pregnancy in diabetic women: results of the Northern Diabetic Pregnancy Audit, 1994. BMJ 315:279-281

18. Khalil A, Syngelaki A, Maiz N et al (2013) Maternal age and adverse pregnancy outcome: a cohort study. Ultrasound Obstet Gynecol 42:634-643

19. Patel EM, Goodnight WH, James AH et al (2015) Temporal trends in maternal medical conditions and stillbirth. Am J Obstet Gynecol 212:673.e1-11

20. Jolly MC, Sebire NJ, Harris JP et al (2003) Risk factors for macrosomia and its clinical consequences: a study of 350,311 pregnancies. Eur J Obstet Gynecol Reprod Biol 111:9-14

21. Cnattingius S, Berne C, Nordström ML (1994) Pregnancy outcome and infant mortality in diabetic patients in Sweden. Diabet Med 11:696-700

22. Reddy UM, Laughon SK, Sun L et al (2010) Prepregnancy risk factors for antepartum stillbirth in the United States. Obstet Gynecol 116:1119-1126

23. Gardosi J, Madurasinghe V, Williams M et al (2013) Maternal and fetal risk factors for stillbirth: population based study. BMJ 346:f108.

24. Anderson KG, Spicer P, Peercy MT (2016) Obesity, Diabetes, and Birth Outcomes Among American Indians and Alaska Natives. Matern Child Health J 20:2548-2556

25. von Kries R, Kimmerle R, Schmidt JE (1997) Pregnancy outcomes in mothers with pregestational diabetes: a population-based study in North Rhine (Germany) from 1988 to 1993. Eur J Pediatr 156:963-967

26. Feig DS, Razzaq A, Sykora K et al (2006) Trends in deliveries, prenatal care, and obstetrical complications in women with pregestational diabetes: a population-based study in Ontario, Canada, 1996-2001. Diabetes Care 29:232-235

27. Mirghani H, Begam M, Bekdache G et al (2012) Specialised fetal and maternal service: outcome of pre-gestational diabetes. J Obstet Gynaecol 32:426-429

28. Macintosh M, Fleming KM, Bailey JA et al (2006) Perinatal mortality and congenital anomalies in babies of women with type 1 or type 2 diabetes in England, Wales, and Northern Ireland: population based study. BMJ 333:177

29. Colatrella A, Braucci S, Festa C et al (2009) Hypertensive disorders in normal/over-weight and obese type 2 diabetic pregnant women. Exp Clin Endocrinol Diabetes 117:373-377

30. Kanda E, Matsuda Y, Makino Y et al (2012) Risk factors associated with altered fetal growth in patients with pregestational diabetes mellitus. J Matern Fetal Neonatal Med 25:1390-1394

31. Al-Biltagi M, Tolba OA, Rowisha MA et al (2015) Speckle tracking and myocardial tissue imaging in infant of diabetic mother with gestational and pregestational diabetes. Pediatr Cardiol 36:445-453

32. Johnstone FD, Nasrat AA, Prescott RJ (1990) The effect of established and gestational diabetes on pregnancy outcome. Br J Obstet Gynaecol 97:1009-1015

33. Stanton SG, Ryerson E, Moore SL et al (2005) Hearing screening outcomes in infants of pregestational diabetic mothers. Am J Audiol 14:86-93

34. Darke J, Glinianaia SV, Marsden P et al (2016) Pregestational diabetes is associated with adverse outcomes in twin pregnancies: a regional register-based study. Acta Obstet Gynecol Scand 95:339-346

35. Loukovaara M, Leinonen P, Teramo K et al (2004) Cord serum glycodelin concentrations in normal pregnancies and pregnancies complicated by diabetes. Arch Gynecol Obstet 270:161-164

36. Leirgul E, Brodwall K, Greve G et al (2016) Maternal Diabetes, Birth Weight, and Neonatal Risk of Congenital Heart Defects in Norway, 1994-2009. Obstet Gynecol 128:1116-1125

37. Egeland GM, Skjaeven R, Irgens L (2002) The reproductive health of daughters of pregestational diabetic women: Medical Birth Registry of Norway. Paediatr Perinat Epidemiol 16:290-296

38. Beyerlein A, von Kries R, Hummel M et al (2010) Improvement in pregnancy-related outcomes in the offspring of diabetic mothers in Bavaria, Germany, during 1987-2007. Diabet Med 27:1379-1384

39. Shefali AK, Kavitha M, Deepa R et al (2006) Pregnancy outcomes in pre-gestational and gestational diabetic women in comparison to non-diabetic women--A prospective study in Asian Indian mothers (CURES-35). J Assoc Physicians India 54:613-618

40. Abdelgadir M, Elbagir M, Eltom A et al (2003) Factors affecting perinatal morbidity and mortality in pregnancies complicated by diabetes mellitus in Sudan. Diabetes Res Clin Pract 60:41-47

41. Evers IM, de Valk HW, Visser GH (2004) Risk of complications of pregnancy in women with type 1 diabetes: nationwide prospective study in the Netherlands. BMJ 328:915

42. Ehrenberg HM, Mercer BM, Catalano PM (2004) The influence of obesity and diabetes on the prevalence of macrosomia. Am J Obstet Gynecol 191:964-968

43. Melamed N, Chen R, Soiberman U et al (2008) Spontaneous and indicated preterm delivery in pregestational diabetes mellitus: etiology and risk factors. Arch Gynecol Obstet 278:129-134

44. Eidem I, Vangen S, Hanssen KF et al (2011) Perinatal and infant mortality in term and preterm births among women with type 1 diabetes. Diabetologia 54:2771-2778

45. Al-Agha R, Firth RG, Byrne M et al (2012) Outcome of pregnancy in type 1 diabetes mellitus (T1DMP): results from combined diabetes-obstetrical clinics in Dublin in three university teaching hospitals (1995-2006). Ir J Med Sci 181:105-109

46. Shand AW, Bell JC, McElduff A et al (2008) Outcomes of pregnancies in women with pre-gestational diabetes mellitus and gestational diabetes mellitus; a population-based study in New South Wales, Australia, 1998-2002. Diabet Med 25:708-715

47. Owens LA, Sedar J, Carmody L et al (2015) Comparing type 1 and type 2 diabetes in pregnancy- similar conditions or is a separate approach required? BMC Pregnancy Childbirth 15:69

48. Wells G, Bleicher K, Han X (2015) Maternal Diabetes, Large-for-Gestational-Age Births, and First Trimester Pregnancy-Associated Plasma Protein-A. J Clin Endocrinol Metab 100:2372-2379

49. Hunt KJ, Marlow NM, Gebregziabher M et al (2012) Impact of maternal diabetes on birthweight is greater in non-Hispanic blacks than in non-Hispanic whites. Diabetologia 55:971-980

50. Kuc S, Wortelboer EJ, Koster MP et al (2011) Prediction of macrosomia at birth in type-1 and 2 diabetic pregnancies with biomarkers of early placentation. BJOG 118:748-754

51. Yves J, Valerie V, Katrien VH et al (2010) Birth weight in type 1 diabetic pregnancy. Obstet Gynecol Int 2010:397623

52. Abell SK, Boyle JA, de Courten B et al (2016) Impact of type 2 diabetes, obesity and glycaemic control on pregnancy outcomes. Aust N Z J Obstet Gynaecol doi: 10.1111/ajo.12521

53. Knight KM, Pressman EK, Hackney DN et al (2012) Perinatal outcomes in type 2 diabetic patients compared with non-diabetic patients matched by body mass index. J Matern Fetal Neonatal Med 25:611-615

54. Oster RT, King M, Morrish DW et al (2014) Diabetes in pregnancy among First Nations women in Alberta, Canada: a retrospective analysis. BMC Pregnancy Childbirth 14:136

55. Sobande AA, Al-Bar H, Archibong EI (2000) Diabetes and perinatal loss. A continuing problem. Saudi Med J 21:161-163

56. Mohsin M, Bauman AE, Jalaludin B (2006) The influence of antenatal and maternal factors on stillbirths and neonatal deaths in New South Wales, Australia. J Biosoc Sci 38:643-657

57. Cundy T, Gamble G, Townend K et al (2000) Perinatal mortality in Type 2 diabetes mellitus. Diabet Med 17:33-39

58. Abell SK, Boyle JA, de Courten B et al (2016) Contemporary type 1 diabetes pregnancy outcomes: impact of obesity and glycaemic control. Med J Aust 205:162-167

59. Achkar M, Dodds L, Giguère Y et al (2015) Vitamin D status in early pregnancy and risk of preeclampsia. Am J Obstet Gynecol 212:511.e1-7

60. Becker T, Vermeulen MJ, Wyatt PR et al (2007) Prepregnancy diabetes and risk of placental vascular disease. Diabetes Care 30:2496-2498

61. Wahabi HA, Esmaeil SA, Fayed A et al (2012) Pre-existing diabetes mellitus and adverse pregnancy outcomes. BMC Res Notes 5:496

62. Macdonald-Wallis C, Silverwood RJ, de Stavola BL et al (2015) Antenatal blood pressure for prediction of pre-eclampsia, preterm birth, and small for gestational age babies: development and validation in two general population cohorts. BMJ 351:h5948

63. Son KH, Lim NK, Lee JW et al (2015) Comparison of maternal morbidity and medical costs during pregnancy and delivery between patients with gestational diabetes and patients with pre-existing diabetes. Diabet Med 32:477-486

64. Jelliffe-Pawlowski LL, Baer RJ, Blumenfeld YJ et al (2015) Maternal characteristics and mid-pregnancy serum biomarkers as risk factors for subtypes of preterm birth. BJOG 122:1484-1493

65. Tolcher MC, Holbert MR, Weaver AL et al (2015) Predicting Cesarean Delivery After Induction of Labor Among Nulliparous Women at Term. Obstet Gynecol 126:1059-1068

66. Vangen S, Stoltenberg C, Holan S et al (2003) Outcome of pregnancy among immigrant women with diabetes. Diabetes Care 26:327-332

67. Wylie BR, Kong J, Kozak SE et al (2002) Normal perinatal mortality in type 1 diabetes mellitus in a series of 300 consecutive pregnancy outcomes. Am J Perinatol 19:169-176

68. Lai FY, Johnson JA, Dover D et al (2016) Outcomes of singleton and twin pregnancies complicated by pre-existing diabetes and gestational diabetes: A population-based study in Alberta, Canada, 2005-11. J Diabetes 8:45-55

69. Penney GC, Mair G, Pearson DW et al (2003) Outcomes of pregnancies in women with type 1 diabetes in Scotland: a national population-based study. BJOG 110:315-318

70. Confidential Enquiry into Maternal and Child Health (2005) Pregnancy in women with type 1 and type 2 diabetes in 2002–2003, England, Wales and Northern Ireland. CEMACH, London

71. Barakat MN, Youssef RM, Al-Lawati JA (2010) Pregnancy outcomes of diabetic women: charting Oman's progress towards the goals of the Saint Vincent Declaration. Ann Saudi Med 30:265-270

72. Lindsay RS, Walker JD, Halsall I et al (2003) Insulin and insulin propeptides at birth in offspring of diabetic mothers. J Clin Endocrinol Metab 88:1664-1671

73. Anderson NH, Sadler LC, Stewart AW et al (2012) Ethnicity, body mass index and risk of pre-eclampsia in a multiethnic New Zealand population. Aust N Z J Obstet Gynaecol 52:552-558

74. Boghossian NS, Yeung E, Mendola P et al (2014) Risk factors differ between recurrent and incident preeclampsia: a hospital-based cohort study. Ann Epidemiol 24:871-7e3

75. Catov JM, Ness RB, Kip KE et al (2007) Risk of early or severe pre-eclampsia related to pre-existing conditions. Int J Epidemiol 36:412-419

76. Dayan N, Pilote L, Opatrny L et al (2015) Combined impact of high body mass index and in vitro fertilization on preeclampsia risk: a hospital-based cohort study. Obesity (Silver Spring) 23:200-206

77. Di Lorenzo G, Ceccarello M, Cecotti V et al (2012) First trimester maternal serum PIGF, free β-hCG, PAPP-A, PP-13, uterine artery Doppler and maternal history for the prediction of preeclampsia. Placenta 33:495-501

78. Goetzinger KR , Singla A , Gerkowicz S , Dicke JM , Gray DL , Odibo AO . Predicting the risk of pre-eclampsia between 11 and 13 weeks' gestation by combining maternal characteristics and serum analytes, PAPP-A and free β-hCG. Prenat Diagn 2010;30 (12-13): 1138-42

79. Lee CJ, Hsieh TT, Chiu TH et al (2000) Risk factors for pre-eclampsia in an Asian population. Int J Gynaecol Obstet 70:327-333

80. Magnussen EB, Vatten LJ, Lund-Nilsen TI et al (2007) Prepregnancy cardiovascular risk factors as predictors of pre-eclampsia: population based cohort study. BMJ 335:978

81. Mahande MJ, Daltveit AK, Mmbaga BT et al (2013) Recurrence of preeclampsia in northern Tanzania: a registry-based cohort study. PLoS One 8:e79116

82. Papageorghiou AT, Yu CK, Erasmus IE (2005) Assessment of risk for the development of pre-eclampsia by maternal characteristics and uterine artery Doppler. BJOG 112:703-709

83. Paré E, Parry S, McElrath TF et al (2014) Clinical risk factors for preeclampsia in the 21st century. Obstet Gynecol 124:763-770

84. Plasencia W, Maiz N, Bonino S et al (2007) Uterine artery Doppler at 11 + 0 to 13 + 6 weeks in the prediction of pre-eclampsia. Ultrasound Obstet Gynecol 30:742-749

85. Poon LC, Kametas NA, Chelemen T et al (2010) Maternal risk factors for hypertensive disorders in pregnancy: a multivariate approach. J Hum Hypertens 24:104-110

86. Rasmussen S, Irgens LM, Albrechtsen S (2000) Predicting preeclampsia in the second pregnancy from low birth weight in the first pregnancy. Obstet Gynecol 96:696-700

87. Sandvik MK, Iversen BM, Irgens LM et al (2010) Are adverse pregnancy outcomes risk factors for development of end-stage renal disease in women with diabetes? Nephrol Dial Transplant 25:3600-3607

88. Sohlberg S, Stephansson O, Cnattingius S et al (2012) Maternal body mass index, height, and risks of preeclampsia. Am J Hypertens 25:120-125

89. Wright D, Akolekar R, Syngelaki A et al (2012) A competing risks model in early screening for preeclampsia. Fetal Diagn Ther 32:171-178

90. Dharan VB, Srinivas SK, Parry S et al (2010) Pregestational diabetes: a risk factor for vaginal birth after cesarean section failure? Am J Perinatol 27:265-270.

91. Günter HH, Scharf A, Hertel H et al (2006) [Perinatal morbidity in pregnancies of women with preconceptional and gestational diabetes mellitus in comparison with pregnancies of non-diabetic women. Results of the perinatal registry of Lower Saxony, Germany]. Z Geburtshilfe Neonatol 210:200-207. [Article in German]

92. Persson M, Pasupathy D, Hanson U et al (2011) Birth size distribution in 3,705 infants born to mothers with type 1 diabetes: a population-based study. Diabetes Care 34:1145-1149

93. Boulot P, Chabbert-Buffet N, d'Ercole C et al (2003) French multicentric survey of outcome of pregnancy in women with pregestational diabetes. Diabetes Care 26:2990-2993

94. Casson IF, Clarke CA, Howard CV et al (1997) Outcomes of pregnancy in insulin dependent diabetic women: results of a five year population cohort study. BMJ 315:275-278

95. Lapolla A, Dalfrà MG, Di Cianni G et al (2008) A multicenter Italian study on pregnancy outcome in women with diabetes. Nutr Metab Cardiovasc Dis 18:291-297

96. Sibai BM, Caritis SN, Hauth JC et al (2000) Preterm delivery in women with pregestational diabetes mellitus or chronic hypertension relative to women with uncomplicated pregnancies. The National institute of Child health and Human Development Maternal- Fetal Medicine Units Network. Am J Obstet Gynecol 183:1520-1524

97. Silva Idos S, Higgins C, Swerdlow AJ et al (2005) Birthweight and other pregnancy outcomes in a cohort of women with pre-gestational insulin-treated diabetes mellitus, Scotland, 1979-95. Diabet Med 22:440-447

98. El Mallah KO, Narchi H, Kulaylat NA et al (1997) Gestational and pre-gestational diabetes: comparison of maternal and fetal characteristics and outcome. Int J Gynaecol Obstet 58:203-209

99. Billionnet C, Mitanchez D, Weill A et al (2017) Gestational diabetes and adverse perinatal outcomes from 716,152 births in France in 2012. Diabetologia doi: 10.1007/s00125-017-4206-6

100. Madan J, Chen M, Goodman E et al (2010) Maternal obesity, gestational hypertension, and preterm delivery. J Matern Fetal Neonatal Med 23:82-88
